# Perioperative safety profile of robotic versus laparoscopic hiatal hernia repair: a systematic review and meta-analysis

**DOI:** 10.3389/fsurg.2026.1993235

**Published:** 2026-10-21

**Authors:** Xingyu Wu, Aokun Zhang, Chao Jin, Haifeng Han, Shuo Yang, Xuefeng Liu, Jinghui Lu

**Affiliations:** 1 Department of General Surgery, Hernia and Abdominal Wall Surgery, Qilu Hospital of Shandong University, Jinan, China; 2 Department of General Surgery, Huantai County People's Hospital, Zibo, Shandong, China

**Keywords:** hiatal hernia, laparoscopy, meta-analysis, minimally invasive surgery, postoperative complications, robotic surgery

## Abstract

**Background:**

Robotic hiatal hernia repair (RHHR) is increasingly used, but whether its technical advantages improve perioperative safety compared with laparoscopic hiatal hernia repair (LHHR) remains uncertain. We compared the short-term safety profiles of RHHR and LHHR, with particular attention to organ-specific postoperative complications.

**Methods:**

MEDLINE (via PubMed), Web of Science, Embase, and the Cochrane Library were searched from inception through 15 July 2025 for comparative studies of RHHR and LHHR in adults. Study selection and data extraction were performed independently by two reviewers. The primary outcome was overall postoperative complications; secondary outcomes included cardiovascular, respiratory, and urological complications, dysphagia, operative time, intraoperative complications, 30-day readmission, mortality, and length of stay. Risk of bias was assessed using the Newcastle–Ottawa Scale for cohort studies, and certainty of evidence was evaluated using GRADE. Odds ratios (ORs) and mean differences (MDs) with 95% confidence intervals (CIs) were pooled as appropriate.

**Results:**

Eleven comparative studies (10 retrospective and one prospective) involving 696,886 patients were included. Overall postoperative complications did not differ significantly between RHHR and LHHR (OR 1.14, 95% CI 0.98–1.33). RHHR was associated with fewer intraoperative complications (OR 0.42, 95% CI 0.24–0.73) and a modestly shorter hospital stay (MD −0.40 days, 95% CI −0.78–−0.02). In contrast, RHHR was associated with higher odds of cardiovascular events (OR 1.24, 95% CI 1.17–1.31), respiratory complications (OR 1.65, 95% CI 1.59–1.71), 30-day readmission (OR 1.23, 95% CI 1.19–1.27), and mortality (OR 1.26, 95% CI 1.11–1.43). No significant differences were observed for urological complications, postoperative dysphagia, or overall operative time. Certainty of evidence ranged from low to very low.

**Conclusions:**

Comparative evidence identified through 15 July 2025 suggests broadly similar overall postoperative morbidity after RHHR and LHHR. RHHR may be associated with fewer intraoperative complications and a shorter hospital stay, but observed associations with higher odds of cardiopulmonary events, readmission, and mortality should be interpreted cautiously because the evidence is predominantly observational and susceptible to residual confounding, case-mix differences, and database-related bias.

**Systematic Review Registration:**

https://www.crd.york.ac.uk/prospero/display_record.php?ID=CRD420251102047, PROSPERO identifier CRD420251102047.

## Introduction

Hiatal hernia is characterized by displacement of the stomach or other abdominal viscera through the esophageal hiatus into the thoracic cavity. The condition has been recognized since the nineteenth century, and the classic anatomic framework distinguishes type I sliding hernias from paraesophageal type II, mixed type III, and type IV hernias involving additional abdominal organs (1–4). Type I sliding hernia is the most common form, and hiatal hernia is closely associated with gastroesophageal reflux disease (GERD) (3–5). Reported prevalence varies widely across populations and diagnostic methods, and larger paraesophageal defects may additionally present with dysphagia, chest discomfort, anemia, obstruction, or cardiopulmonary symptoms (6).

Surgical management has evolved from open repair to minimally invasive laparoscopic and robotic approaches. Laparoscopic repair remains an established standard because it reduces surgical trauma and facilitates recovery compared with open surgery (7). Robotic platforms provide three-dimensional visualization, wristed instrumentation, tremor filtration, and improved ergonomics, features that may be advantageous during mediastinal dissection, crural closure, and complex or redo repairs (8–10). Robotic-assisted surgery has consequently been adopted increasingly in Europe, the United States, and other high-volume centers. These technical benefits must, however, be weighed against setup and docking time, costs, institutional experience, and the possibility that robotic surgery is preferentially selected for larger or more complex hernias (11–13).

Previous comparative studies have reported both favorable and unfavorable perioperative findings for robotic repair, and consensus regarding its clinical value remains incomplete. Several systematic reviews have compared robotic and laparoscopic hiatal hernia or anti-reflux surgery, but their conclusions have varied with the populations, study periods, and outcome definitions included (14–18). Recent syntheses generally suggest comparable overall perioperative outcomes, while differences in operative time, cost, length of stay, and selected complications remain inconsistent. Because an aggregate complication endpoint may obscure clinically relevant differences between organ systems, a focused assessment of perioperative safety, including cardiovascular and respiratory events, remains informative.

This systematic review and meta-analysis therefore compared robotic hiatal hernia repair (RHHR) with laparoscopic hiatal hernia repair (LHHR) in adults. Overall postoperative morbidity was designated as the primary outcome, while organ-specific complications, postoperative dysphagia, operative time, conversion, intraoperative complications, 30-day readmission, mortality, and length of stay were evaluated as secondary outcomes.

## Methods

### Protocol and reporting standard

This systematic review and meta-analysis was conducted in accordance with the Preferred Reporting Items for Systematic Reviews and Meta-Analyses (PRISMA) 2020 statement (19). The protocol was registered prospectively in PROSPERO (CRD420251102047) before data extraction.

### Eligibility criteria

Eligibility was defined *a priori* using the population, intervention, comparator, outcomes, and study design (PICOS) framework. Population: adults (≥18 years) undergoing surgical repair of hiatal or paraesophageal hernia. Intervention: robotic-assisted hiatal hernia repair (RHHR). Comparator: conventional laparoscopic hiatal hernia repair (LHHR). Outcomes: at least one prespecified perioperative outcome, including operative time, conversion, intraoperative complications, overall postoperative complications, cardiovascular complications, respiratory complications, urological complications, postoperative dysphagia, 30-day readmission, mortality, or length of stay. Study designs: randomized controlled trials and comparative observational studies published from 1 January 2010 through 15 July 2025.

Studies were excluded if they were non-English publications, involved pediatric populations or populations restricted to patients with obesity, did not concern hiatal hernia repair, evaluated non-comparable techniques, did not report robotic and laparoscopic outcomes separately, or provided no quantitative data for any prespecified outcome. Meta-analyses, reviews, conference abstracts, editorials, commentaries, case reports, non-comparative studies, and health technology assessments not published in peer-reviewed journals were also excluded.

Studies reporting extractable quantitative comparative data for at least one prespecified outcome remained eligible even if they did not report other secondary outcomes. The population-based exclusion applied to reports restricted to patients with obesity, not to individual patients with obesity within otherwise eligible mixed cohorts.

### Information sources and search strategy

Two investigators independently searched MEDLINE (via PubMed), Web of Science, Embase, and the Cochrane Library from inception through 15 July 2025. The PubMed search strategy was: ((“hernia, hiatal”[MeSH Terms]) OR (“Herni*, Hiat*”[Title/Abstract]) OR (“Hiat* Herni*”[Title/Abstract]) OR (“Paraesophageal Hiatal Hernia*”[Title/Abstract]) OR (“Sliding Hiatal Hernia*”[Title/Abstract]) OR (“Paraesophageal Hernia*”[Title/Abstract]) OR (“Sliding Esophageal Hernia”[Title/Abstract]) OR (“Esophageal Hernia*”[Title/Abstract]) OR (“Hernia*, Esophageal”[Title/Abstract])) AND ((“Robotic Surgical Procedures”[MeSH Terms]) OR (“Robotic Surgical Procedure*”[Title/Abstract]) OR (“Robotic-Assisted Surger*”[Title/Abstract]) OR (“Robot-Assisted Surger*”[Title/Abstract]) OR (“Robot Surger*”[Title/Abstract]) OR (“Robot-Enhanced Procedure*”[Title/Abstract])). Equivalent syntax was adapted for Web of Science, Embase, and the Cochrane Library. The complete database-specific strategies are provided in the Supplementary Material.

#### Database-specific search strategies (Supplementary Material 2)

The PubMed strategy is presented above. The Embase strategy comprised the two archived concept blocks combined with AND. The Web of Science and Cochrane Library expressions presented below were verified by the authors during revision; this revision-stage verification was separate from the original database searches.

#### Embase.com

#1 “hiatal hernias”:ti,ab,kw OR “hiatus hernia”:ti,ab,kw OR “sliding esophageal hernia”:ti,ab,kw OR “esophageal hernia”:ti,ab,kw OR “hernia, esophageal”:ti,ab,kw OR “hiatus hernia”/exp

#2 “surgical procedures, robotic”:ti,ab,kw OR “robot-enhanced procedures”:ti,ab,kw OR “robot surgery”:ti,ab,kw OR “robotic-assisted surgeries”:ti,ab,kw OR “procedure, robotic surgical”:ti,ab,kw OR “robot assisted surgery”/exp

#3 #1 AND #2

#### Web of science

TS = (“hiatal hernia” OR “herni* hiat*” OR “hiat* herni*” OR “paraesophageal hiatal hernia*” OR “sliding hiatal hernia*” OR “paraesophageal hernia*” OR “sliding esophageal hernia” OR “esophageal hernia*” OR “hernia* esophageal”) AND TS = (robot*)

#### Cochrane library: search manager

#1 MeSH descriptor: [Hernia, Hiatal] explode all trees

#2 ((herni* NEXT hiat*) OR (hiat* NEXT herni*) OR (paraesophageal NEXT hiatal NEXT hernia*) OR (sliding NEXT hiatal NEXT hernia*) OR (paraesophageal NEXT hernia*) OR (sliding NEXT esophageal NEXT hernia) OR (esophageal NEXT hernia*) OR (hernia* NEXT esophageal)):ti,ab,kw

#3 MeSH descriptor: [Robotic Surgical Procedures] explode all trees

#4 ((robotic NEXT surgical NEXT procedure*) OR (robotic NEXT assisted NEXT surger*) OR (robot NEXT assisted NEXT surger*) OR (robot NEXT surger*) OR (robot NEXT enhanced NEXT procedure*)):ti,ab,kw

#5 (#1 OR #2) AND (#3 OR #4)

### Study selection

Two reviewers independently screened titles and abstracts and assessed the available full-text reports against the eligibility criteria. Disagreements were resolved by discussion and, when necessary, adjudication by a senior third reviewer. Reports that could not be retrieved were recorded separately from reports excluded after full-text assessment. The reasons for full-text exclusion are summarized in the PRISMA flow diagram (Figure 1).

**Figure 1:**
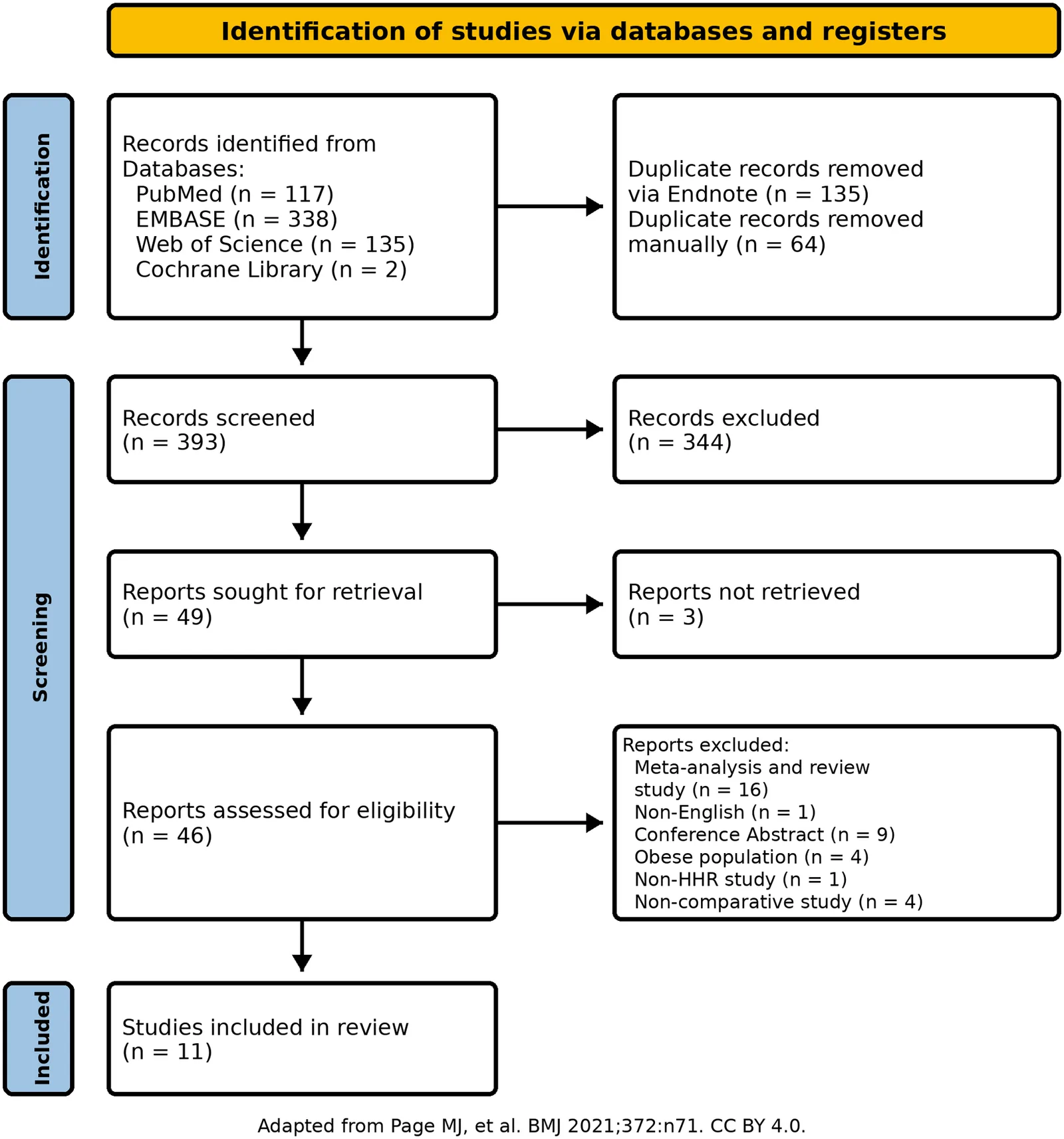
PRISMA 2020 flow diagram of study identification and selection.

### Data extraction

Two reviewers independently extracted data using a standardized form. Extracted variables included study design, country, study period, data source or setting, sample size, demographic characteristics, body mass index (BMI), hernia size and type, comorbidities, operative details (including fundoplication technique, mesh use, conversion, and esophageal lengthening when reported), and prespecified short-term outcomes. Outcomes included intraoperative complications; overall morbidity; mortality; 30-day readmission; postoperative dysphagia; cardiovascular, urological, and respiratory complications; operative time; and length of stay. When multiple reports used potentially overlapping databases and study periods, the possibility of duplicate populations was assessed before pooling. Study authors were contacted when essential data were unclear or unavailable.

Four studies used large databases or registries: the Nationwide Readmissions Database (NRD; Klock et al., 2010–2019), TriNetX (Kooiker et al., 2013–2022), the National Inpatient Sample (NIS; Ward et al., 2010–2015), and ACS-NSQIP (Powell et al., 2022) (20–23). The NRD and NIS covered overlapping periods and potentially overlapping hospital populations, and some institutions may have contributed to more than one database. Because the published aggregate data did not permit cross-database patient linkage, population overlap could not be quantified or excluded. Analyses omitting individual databases or all large database studies therefore assessed the influence of data source; they did not establish patient-level independence.

### Outcome definitions

The primary outcome was overall postoperative complications. Because operational definitions varied across single-center cohorts and administrative or registry databases, the definition reported by each primary study was retained rather than assuming a uniform Clavien–Dindo classification (24). Secondary outcomes were postoperative cardiovascular events, respiratory complications, urological complications, postoperative dysphagia, operative time, intraoperative complications, conversion, 30-day readmission, mortality, and length of stay. Organ-specific outcomes were analyzed as distinct secondary outcomes and were not treated as subgroups of the overall complication outcome.

Study-specific outcome definitions and observation windows are summarized in Supplementary Table S2. The table distinguishes in-hospital and 30-day outcomes from symptoms assessed during longer-term follow-up. Non-reporting was distinguished from an explicitly reported zero-event result. The interpretation of composite outcomes also depends on whether the reported numerator represents patients with at least one complication or potentially overlapping component events.

### Risk of bias assessment

Comparative observational studies were assessed using the Newcastle–Ottawa Scale (NOS) for cohort studies, covering cohort selection, comparability, and outcome assessment (25, 26). Two reviewers independently assessed each study, with disagreements resolved by consensus. The maximum NOS score was nine stars; higher scores indicated lower risk of bias.

The cohort NOS comprises four selection items, one comparability item with a maximum of two stars, and three outcome-assessment items (26). Supplementary Table S3 presents the original ratings together with item-level scoring guidance and an evidence-recording framework. Selection addresses cohort representativeness, the source of the comparison cohort, ascertainment of the surgical approach, and whether the assessed outcome was absent at baseline. Comparability is based on documented matching or statistical adjustment for important baseline confounders, rather than nonsignificant differences in baseline characteristics. The factors controlled in each primary study and the matched or unmatched data actually used in this review are documented separately.

Outcome assessment considers the method of ascertainment, the adequacy of the observation window, and the completeness of follow-up as separate items. Follow-up requirements are interpreted in relation to the outcome: observation to discharge applies to explicitly in-hospital outcomes, whereas a 30-day endpoint requires an adequate 30-day observation window. The scoring guidance was expanded during revision to facilitate verification of the submitted ratings.

### Certainty of evidence

The certainty of evidence for each pooled outcome was assessed using the GRADE framework, considering risk of bias, inconsistency, indirectness, imprecision, and publication bias. Because the evidence base was observational, certainty was initially rated as low and was downgraded or upgraded only when justified. The detailed GRADE summary of findings is provided in Supplementary Table S1.

### Statistical analysis

For dichotomous outcomes, pooled effects were expressed as odds ratios (ORs) with 95% CIs using Mantel–Haenszel methods. Continuous outcomes were summarized as mean differences (MDs) with 95% CIs using inverse-variance methods. Statistical heterogeneity was evaluated using Cochran's *Q* test and the I² statistic, with I² > 50% considered substantial heterogeneity. Random-effects models were used when heterogeneity was substantial; otherwise, fixed-effect models were used. Sensitivity and subgroup analyses were undertaken to explore substantial heterogeneity where data permitted. Forest plots were used to display pooled estimates. Analyses were performed using Review Manager and Stata.

The denominator for each outcome included only participants from studies that contributed usable data for that outcome. Consequently, denominators differed across outcomes. An outcome that was not reported was distinguished from an explicitly reported zero-event outcome.

#### Sensitivity analyses

Post hoc sensitivity analyses were restricted to the four outcomes with I² > 50% in the primary analysis: overall postoperative complications (I² = 86%; 11 studies), urological complications (I² = 63%; five studies), operative time (I² = 93%; six studies), and length of stay (I² = 97%; seven studies). Outcomes were selected according to their heterogeneity in the primary analysis, before any study was excluded. All resulting analyses were retained, including those in which I² subsequently fell to 50% or below.

For each eligible outcome, leave-one-out analyses sequentially excluded each contributing study. Additional analyses excluded studies with original NOS scores ≤6, as reported in Table 4. Where relevant, the NRD and NIS studies were omitted separately and together, and all large database or registry studies were excluded. Each reduced dataset was analyzed using the same model-selection rule as the primary analysis: DerSimonian–Laird random effects for I² > 50%, and Mantel–Haenszel fixed effects for binary outcomes or inverse-variance fixed effects for continuous outcomes otherwise. The fixed-effect operative-time analysis after exclusion of Gerull et al. was therefore retained. These supplementary analyses were implemented in Python with NumPy and SciPy and were interpreted as exploratory; no multiplicity adjustment was applied.

An exploratory univariable meta-regression examined whether the estimated association between surgical approach and overall postoperative complications differed between large database or registry studies and other studies (four versus seven studies). Residual between-study variance was estimated by restricted maximum likelihood, with a modified Hartung–Knapp covariance adjustment (Supplementary Table S6). Meta-regression was not performed for the other three high-heterogeneity outcomes because only five to seven studies contributed to each. These study-level associations were interpreted as exploratory and not as evidence of patient-level causation (27).

#### Exploratory sequential information assessment

A *post hoc* information-based trial sequential assessment was performed for overall postoperative complications only (28–30). Studies were ordered by publication year and then by the first author's surname within each year. Two-sided Lan–DeMets/O'Brien–Fleming efficacy boundaries were calculated using an overall alpha of 0.05. Target ORs of 0.90 and 0.80 were examined as illustrative assumptions, rather than established minimum clinically important differences. The first scenario served as the principal information assessment, and the second assessed dependence on the assumed effect size; the scenarios were not interpreted as independent confirmatory tests. The alternative effects were symmetric on the log-OR scale. Study-level log ORs and nominal variances were taken from the existing supplementary analysis data. Statistical information was calculated as the sum of inverse-variance weights, with between-study variance fixed at the previously estimated value of 0.026451; the primary analyses were not replaced.

Nonbinding symmetric futility boundaries were added using O'Brien–Fleming-type beta spending. The required maximum information level was determined numerically to obtain an 80% probability of crossing an efficacy boundary in the correct direction under each target effect when the specified stopping rules were followed. Efficacy boundaries were calculated without assuming futility stopping, preserving nominal type-I error control if futility was ignored. Calibration used the observed information times and one planning-only terminal assessment at the required maximum information; this terminal assessment included no additional observed study or outcome. Repeated 95% confidence intervals were obtained by applying the stage-specific efficacy boundary to the cumulative standard error. Numerical integration was checked at two quadrature resolutions and against independent Gaussian-increment simulations of 500,000 sequences per scenario. The algorithms, assumptions, parameters, and numerical checks are provided with Supplementary Table S7.

The assessment was implemented in Python with NumPy and SciPy. Calibration and coverage are conditional on a model with independent Gaussian score increments and fixed nuisance parameters; they do not establish prospective power for this retrospective dataset. Survey-weighted national counts, potential population overlap, and unresolved composite-outcome counting limit the clinical interpretation. The first efficacy-boundary crossing was recorded; subsequent observations were displayed retrospectively and did not negate the initial crossing. Entering a futility region would not establish equivalence, and neither a boundary crossing nor the attainment of an information target removes systematic bias.

## Results

### Study selection and characteristics

The database searches identified 592 records: 117 from PubMed, 338 from Embase, 135 from Web of Science, and 2 from the Cochrane Library. After 199 duplicates were removed (135 using EndNote and 64 manually), 393 records underwent title and abstract screening, of which 344 were excluded. Full texts were sought for the remaining 49 reports; 3 could not be retrieved, leaving 46 reports for full-text eligibility assessment. Of these, 35 were excluded: meta-analyses or reviews (*n* = 16), a non-English publication (*n* = 1), conference abstracts (*n* = 9), studies restricted to patients with obesity (*n* = 4), a study not concerning hiatal hernia repair (*n* = 1), and non-comparative studies (*n* = 4). The remaining 11 studies were included in the systematic review and meta-analysis (Figure 1).

Eleven comparative studies published between 2013 and 2025, involving 696,886 patients, were included (20–23, 31–37): 73,220 patients underwent RHHR and 623,666 underwent LHHR. Two studies were from Germany (31, 32), one from Switzerland (36), one from Japan (37), and seven from the United States (20–23, 33–35). Ten studies were retrospective comparative cohorts, whereas the RATHER study by Wilhelm et al. was prospective and comparative (36). Seven studies were single-center cohorts (31–37), and four used large databases or registries, including the Nationwide Readmissions Database, TriNetX, the HCUP National Inpatient Sample, and ACS-NSQIP (20–23). Patient characteristics, hernia size and type, and operative details are summarized in Tables 1–3.

**Table 1:** Characteristics of included studies.

Study	Country	Study period	Study design	Patients (RHHR/LHHR)	Female (%)		Age (years)		Follow-up (months)		BMI (kg/m²)		Comorbidities	
					LHHR	RHHR	LHHR	RHHR	LHHR	RHHR	LHHR	RHHR	LHHR	RHHR
Benedix et al. (31)	Germany	2016–2020	Retrospective	55/85	65.9	67.3	62.9 ± 11.6^b^	63.5 ± 12.3^b^	3	3	29.3 ± 3.8^b^	29.5 ± 4.4^b^	P, C, H, T2DM, R, HE, BE, G, S	P, C, H, T2DM, R, HE, BE, G, S
Gehrig et al. (32)	Germany	2003–2007	Retrospective	12/17	29.4	75	60.2 ± 11.8^b^	68.1 ± 7.9^b^	NR	NR	26.6 ± 4.4^b^	25.7 ± 2.6^b^	NR	NR
Gerull et al. (33)	USA	2009–2019	Retrospective	830/1,024	67.1	64.7	63.1 ± 14.2^b^	65.1 ± 13.1^b^	NR	NR	NR	NR	NR	NR
Klock et al. (20)	USA	2010–2019	Retrospective NRD	58,650/459,214	79.8	78.1	53 (42–64)^a^	57 (45–68)^a^	NR	NR	NR	NR	CAD, COPD, CHF, O, RF, SA	CAD, COPD, CHF, O, RF, SA
Kooiker et al. (21)	USA	2013–2022	Retrospective TriNetX	1,514/1,514	72.1	70.7	63.6 ± 13.1^b^	63.3 ± 12.9^b^	NR	NR	29.6 ± 5.8^b^	29.5 ± 5.4^b^	IHD, H, DM, COPD, SA, CKD, PVD, HF	IHD, H, DM, COPD, SA, CKD, PVD, HF
Lekarczyk et al. (34)	USA	2020–2021	Retrospective	31/42	85.7	74.2	65 (57–69)^a^	68 (58–75)^a^	NR	NR	30.0 ± 4.2^b^	27.4 ± 4.6^b^	H, CAD, CVA, DM, HF, MVD, PFO	H, CAD, CVA, DM, HF, MVD, PFO
Soliman et al. (35)	USA	2012–2017	Retrospective	142/151	73.5	71.8	63 (54–73)^a^	61 (48–72)^a^	NR	NR	29.3 (25.4–33.3)^a^	29.7 (26.5–33.3)^a^	H, CAD, PVD, DM, COPD	H, CAD, PVD, DM, COPD
Wilhelm et al. (36)	Switzerland	2015–2019	Prospective comparative	36/19	74	64	76 (44–91)^a^	71 (44–90)^a^	27 (8–53)^a^	31 (13–46)^a^	28 (17.4–42.8)^a^	29.7 (22.3–35.8)^a^	NR	NR
Ward et al. (22)	USA	2010–2015	Retrospective HCUP-NIS	9,897/158,432	77.3	76	54.9 ± 15.7^b^	59.1 ± 15.2^b^	NR	NR	NR	NR	H, DM, O, RF, CAD, PVD, COPD, SA	H, DM, O, RF, CAD, PVD, COPD, SA
Powell et al. (23)	USA	2022	Retrospective NSQIP	1,567/2,778	72.4	74.5	66 (56–73)^a^	64 (53–73)^a^	NR	NR	29.9 (26.5–33.5)^a^	30.2 (27.0–34.1)^a^	H, HF, COPD, DM, R, BD	H, HF, COPD, DM, R, BD
Ueda et al. (37)	Japan	2000–2023	Retrospective	486/390	70.5	69.1	63.2 ± 12.7^b^	61.9 ± 13.6^b^	NR	NR	28.9 ± 4.5^b^	29.2 ± 4.7^b^	NR	NR

RHHR, robotic hiatal hernia repair; LHHR, laparoscopic hiatal hernia repair; BMI, body mass index; NR, not reported; NRD, Nationwide Readmissions Database; HCUP, Healthcare Cost and Utilization Project; NIS, National Inpatient Sample; NSQIP, National Surgical Quality Improvement Program. Comorbidity and history abbreviations: BD, bleeding disorder; BE, Barrett's esophagus; C, cardiac disease; CAD, coronary artery disease; CHF, congestive heart failure; CKD, chronic kidney disease; COPD, chronic obstructive pulmonary disease; CVA, cerebrovascular accident; DM, diabetes mellitus; G, gastritis; H, hypertension; HE, hepatic disease; HF, heart failure; IHD, ischemic heart disease; MVD, major vascular disease; O, obesity; P, pulmonary disease; PFO, previous foregut operation; PVD, peripheral vascular disease; R, renal disease; RF, renal failure; S, smoking; SA, sleep apnea; T2DM, type 2 diabetes mellitus.

a median (interval as reported in the original study).

b mean ± standard deviation.

**Table 2:** Hiatal hernia size and type reported in included studies.

Study	Hiatal hernia size (n)		Type I (n)		Type II (n)		Type III (n)		Type IV (n)	
	LHHR	RHHR	LHHR	RHHR	LHHR	RHHR	LHHR	RHHR	LHHR	RHHR
Benedix et al. (31)	< 5 cm: (47); 5–10 cm: (13); > 10 cm: (25)	< 5 cm: (28); 5–10 cm: (17); > 10 cm: (10)	NR	NR	NR	NR	NR	NR	NR	NR
Gehrig et al. (32)	NR	NR	NR	NR	NR	NR	NR	NR	NR	NR
Gerull et al. (33)	NR	NR	0	0	12	15	845	659	167	156
Klock et al. (20)	NR	NR	NR	NR	NR	NR	NR	NR	NR	NR
Kooiker et al. (21)	NR	NR	NR	NR	NR	NR	NR	NR	NR	NR
Lekarczyk et al. (34)	NR	NR	4	4	8	2	29	23	1	2
Soliman et al. (35)	NR	NR	37	50	0	0	109	87	5	5
Wilhelm et al. (36)	NR	NR	0	0	0	0	0	4	19	32
Ward et al. (22)	NR	NR	NR	NR	NR	NR	NR	NR	NR	NR
Powell et al. (23)	NR	NR	NR	NR	NR	NR	NR	NR	NR	NR
Ueda et al. (37)	NR	NR	116	197	4	4	249	258	9	20

NR, not reported.

**Table 3:** Operative characteristics reported in included studies.

Study	RHHR								LHHR							
	Nissen	Toupet	Dor	Hill	LINX	Mesh used	Conversion to open	Esophageal lengthening (Collis)	Nissen	Toupet	Dor	Hill	LINX	Mesh used	Conversion to open	Esophageal lengthening (Collis)
Benedix et al. (31)	0	55	0	NR	0	30	1	NR	0	85	0	NR	0	55	2	NR
Gehrig et al. (32)	6	NR	NR	NR	NR	5	0	NR	9	NR	NR	NR	NR	7	1	NR
Gerull et al. (33)	NR	NR	NR	NR	NR	NR	0	1	NR	NR	NR	NR	NR	NR	72	113
Klock et al. (20)	NR	NR	NR	NR	NR	NR	NR	NR	NR	NR	NR	NR	NR	NR	NR	NR
Kooiker et al. (21)	NR	NR	NR	NR	NR	NR	NR	NR	NR	NR	NR	NR	NR	NR	NR	NR
Lekarczyk et al. (34)	NR	NR	NR	NR	NR	NR	NR	NR	NR	NR	NR	NR	NR	NR	NR	NR
Soliman et al. (35)	26	63	1	NR	52	2	1	NR	23	126	2	NR	0	2	0	NR
Wilhelm et al. (36)	0	29	0	NR	0	36	0	NR	4	15	0	NR	0	10	0	NR
Ward et al. (22)	NR	NR	NR	NR	NR	NR	NR	NR	NR	NR	NR	NR	NR	NR	NR	NR
Powell et al. (23)	NR	NR	NR	NR	NR	556	NR	NR	NR	NR	NR	NR	NR	1,008	NR	NR
Ueda et al. (37)	46	305	126	3	NR	NR	4	0	277	83	19	9	NR	NR	6	0

NR, not reported.

**Table 4:** Quality assessment of included cohort studies using the Newcastle–Ottawa Scale (NOS).

Study	Selection				Comparability	Outcome			Total score
	Representativeness of exposed cohort	Selection of non-exposed cohort	Ascertainment of exposure	Outcome absent at start		Assessment of outcome	Follow-up long enough	Adequacy of follow-up	
Benedix et al. (31)	★	★	★	★	★★	★	★	-	8
Gehrig et al. (32)	★	★	★	★	-	★	★	-	6
Gerull et al. (33)	★	★	★	★	-	★	★	-	6
Klock et al. (20)	★	★	★	★	★★	★	★	-	8
Kooiker et al. (21)	★	★	★	★	★★	★	★	-	8
Lekarczyk et al. (34)	★	★	★	★	-	★	★	-	6
Soliman et al. (35)	★	★	★	★	★	★	★	-	7
Wilhelm et al. (36)	★	★	★	★	-	★	★	★	7
Ward et al. (22)	★	★	★	★	★★	★	★	-	8
Powell et al. (23)	★	★	★	★	★★	★	★	-	8
Ueda et al. (37)	★	★	★	★	-	★	★	-	6

★, criterion fulfilled; -, criterion not fulfilled or insufficient information. Selection and Outcome items contribute a maximum of one star each, whereas Comparability contributes a maximum of two stars. The maximum NOS score is 9. NOS, Newcastle–Ottawa Scale.

### Risk of bias

Using the NOS cohort version, the included studies had total scores ranging from 6 to 8 stars (Table 4), indicating variable methodological quality, with limitations mainly related to between-group comparability and adequacy of follow-up. GRADE certainty ranged from low to very low across the pooled outcomes (Supplementary Table S1), reflecting the observational evidence base, residual confounding, inconsistency, and imprecision for several outcomes.

According to the original NOS ratings, Gehrig, Gerull, Lekarczyk, and Ueda had scores ≤6. Supplementary Table S3 provides the scoring guidance and evidence fields for verification. The NOS-restricted analyses used the studies identified by these original scores; a score of ≤6 was treated as a review-specific sensitivity threshold rather than a universal NOS definition of high risk of bias.

### Primary outcome: overall postoperative complications

All 11 studies reported overall postoperative complications (20–23, 31–37). Complications occurred in 10.44% (7,646/73,220) of patients in the RHHR group and 8.02% (49,993/623,666) in the LHHR group. The pooled analysis showed no statistically significant difference between approaches (OR 1.14, 95% CI 0.98–1.33; *p* = 0.10; I² = 86%) (Figure 2).

**Figure 2:**
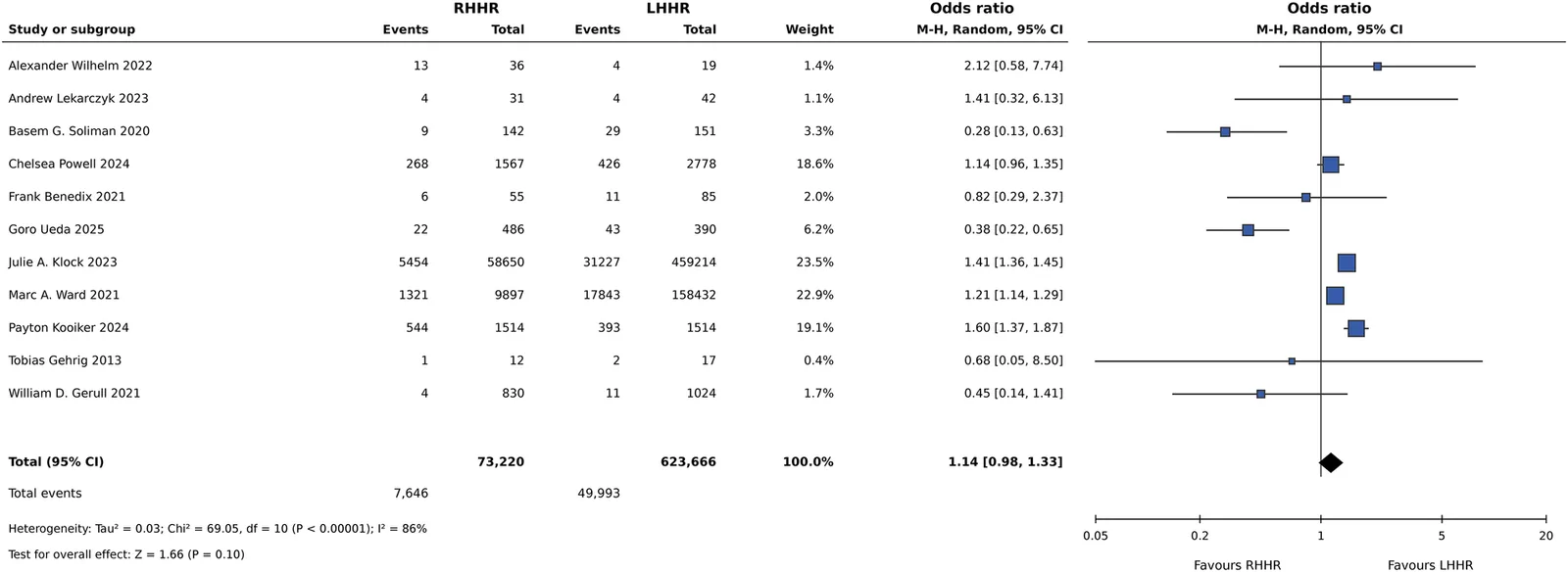
Forest plot of overall postoperative complications.

### Secondary outcomes

#### Postoperative cardiovascular events

All included studies reported cardiovascular events (20–23, 31–37). Events occurred in 1.90% (1,391/73,220) of RHHR patients and 1.87% (11,907/623,666) of LHHR patients. The pooled estimate favored LHHR (OR 1.24, 95% CI 1.17–1.31; *p* < 0.001; I² = 15%) (Figure 3). Although statistically significant, the absolute difference in event rates was small and should be interpreted in the context of the very large database-derived sample.

**Figure 3:**
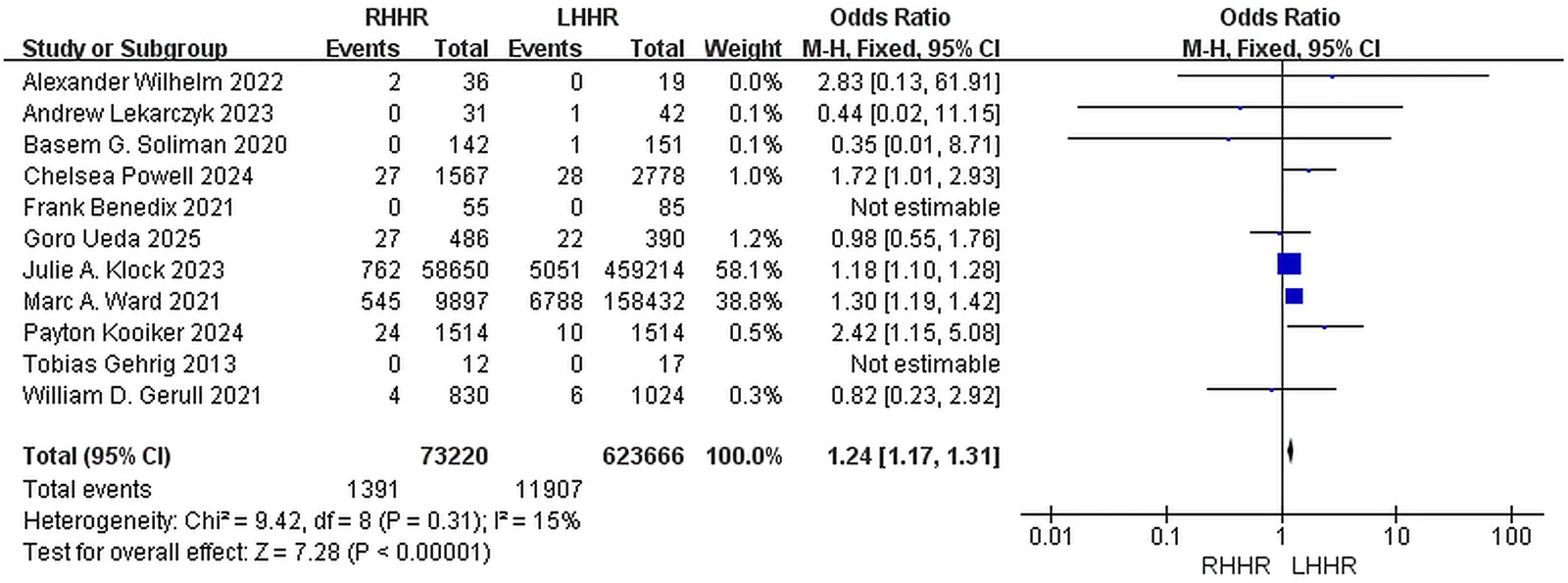
Forest plot of postoperative cardiovascular events.

#### Postoperative respiratory complications

Nine studies reported respiratory complications (20–23, 31, 32, 34–36). Respiratory complications occurred in 5.06% (3,636/71,904) of RHHR patients and 2.93% (18,211/622,252) of LHHR patients. RHHR was associated with higher odds of respiratory complications (OR 1.65, 95% CI 1.59–1.71; *p* < 0.001; I² = 48%) (Figure 4).

**Figure 4:**
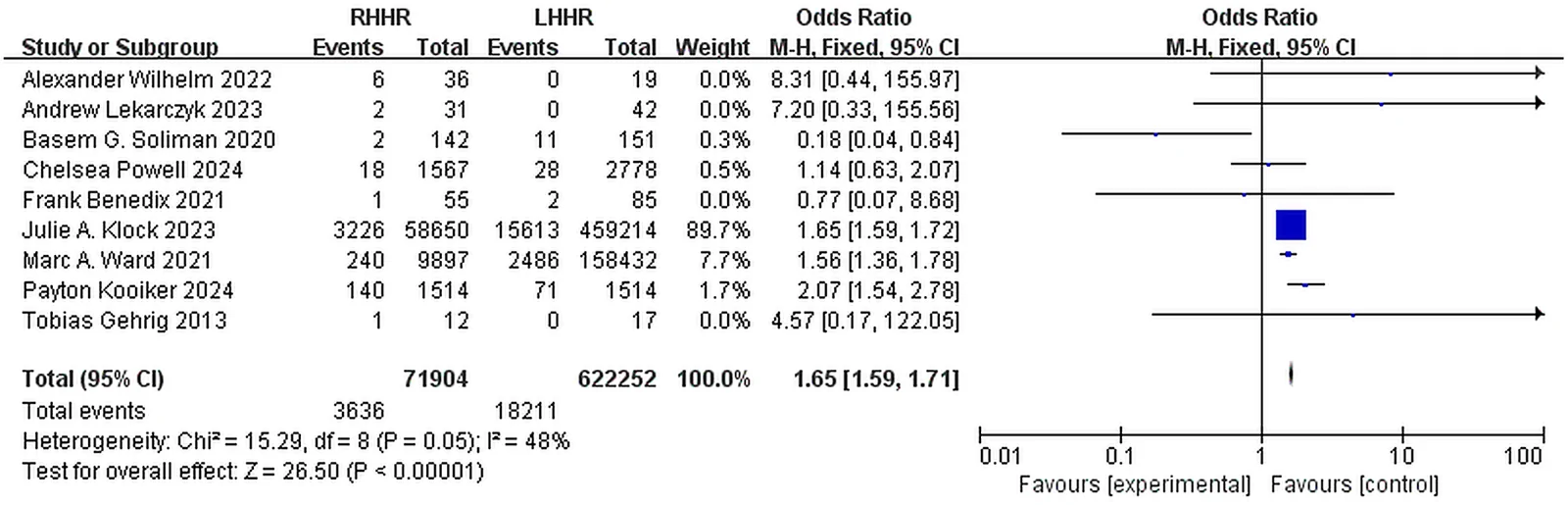
Forest plot of postoperative respiratory complications.

#### Postoperative urological complications

Five studies reported urological complications (20, 21, 23, 35, 36). Events occurred in 2.41% (1,495/61,909) of RHHR patients and 1.70% (7,861/463,676) of LHHR patients. The pooled difference was not statistically significant (OR 1.39, 95% CI 0.88–2.17; *p* = 0.16; I² = 63%) (Figure 5).

**Figure 5:**
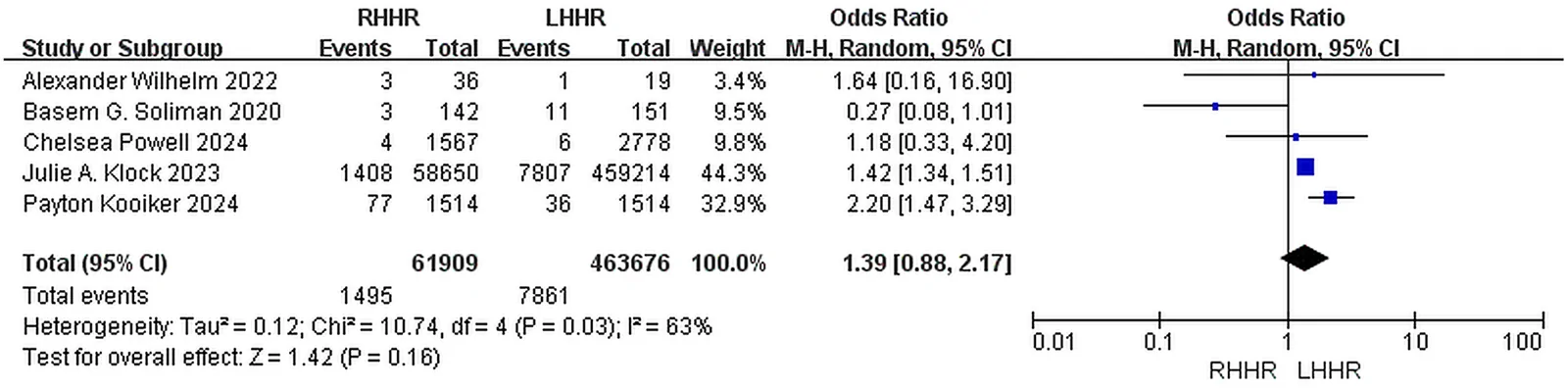
Forest plot of postoperative urological complications.

#### Postoperative dysphagia

Three studies reported postoperative dysphagia (31, 36, 37). Dysphagia occurred in 10.31% (56/543) of RHHR patients and 8.68% (42/484) of LHHR patients. No statistically significant difference was observed (OR 1.23, 95% CI 0.81–1.88; *p* = 0.34; I² = 0%) (Figure 6).

**Figure 6:**
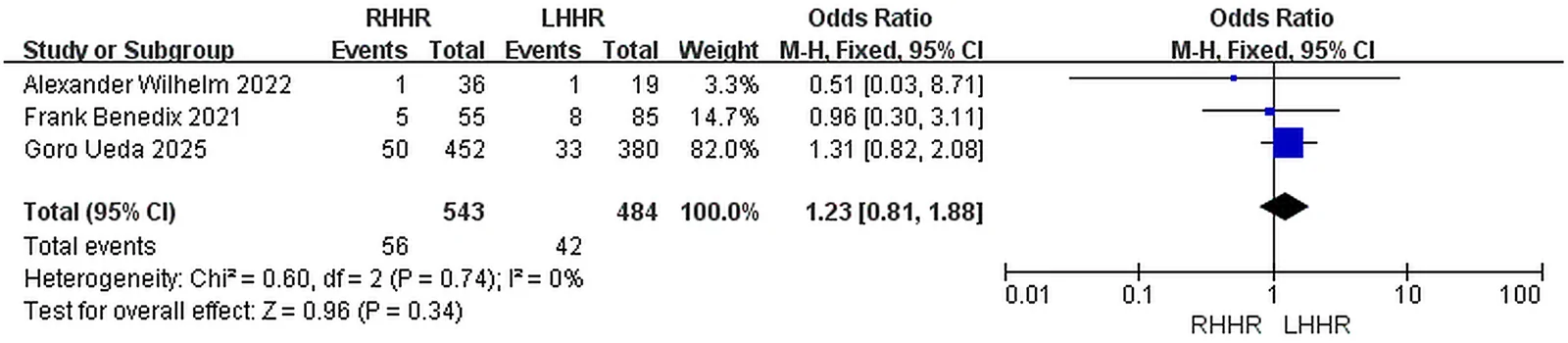
Forest plot of postoperative dysphagia.

#### Operative time

Six studies involving 3,255 patients reported operative time (31–35, 37). The overall pooled estimate did not show a statistically significant difference between RHHR and LHHR (MD 12.24 min, 95% CI −7.10–31.58; *p* = 0.21; I² = 93%) (Figure 7A). Leave-one-out sensitivity analysis identified the study by Gerull et al. (33) as an important contributor to heterogeneity (Figure 7B). After excluding that study, RHHR was associated with a longer operative time (MD 22.95 min, 95% CI 16.71–29.19; *p* < 0.001), while heterogeneity decreased to I² = 40% (Figure 7C).

**Figure 7:**
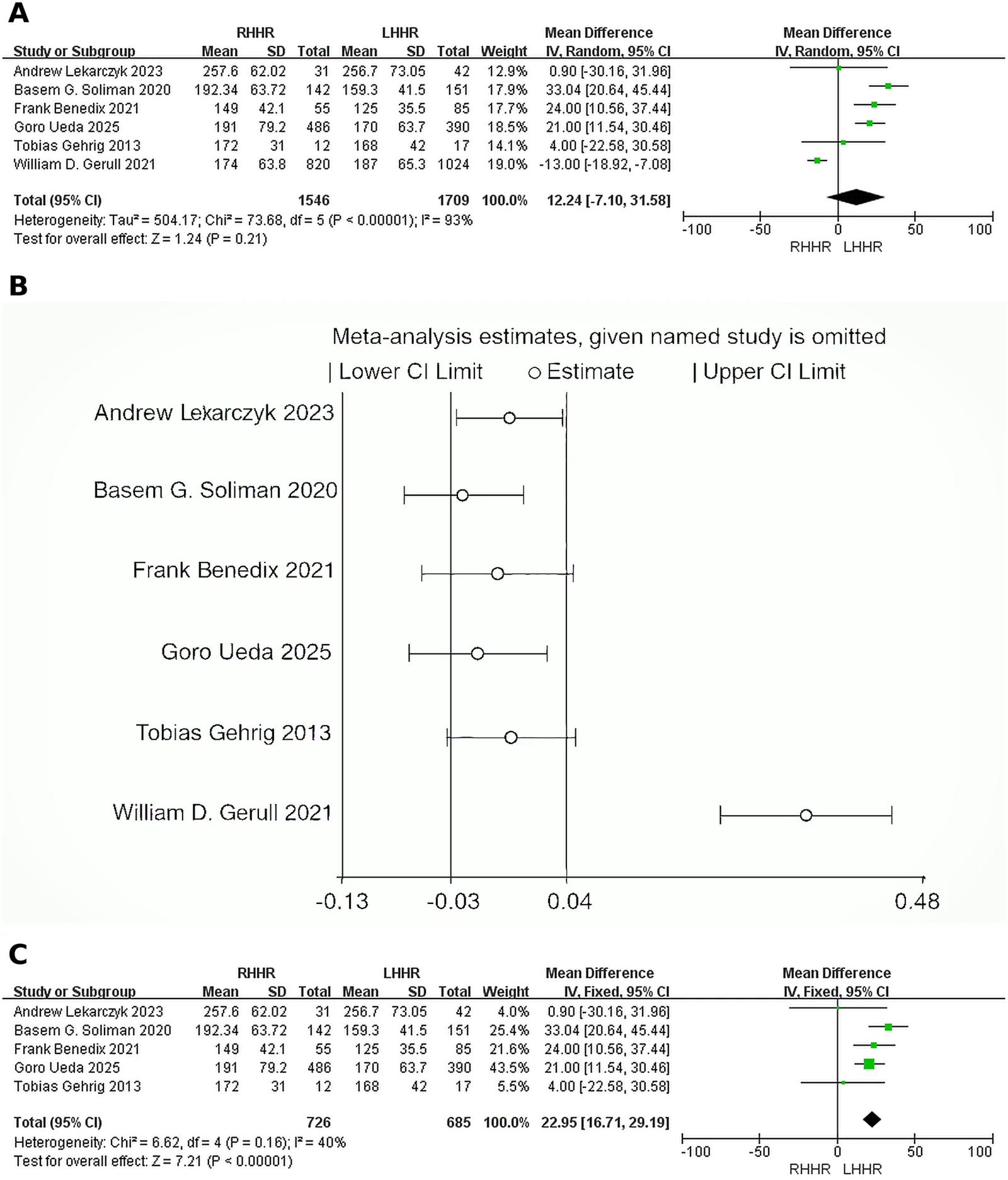
Operative time analyses. **(A)** Overall pooled operative time; **(B)** leave-one-out sensitivity analysis; **(C)** pooled operative time after exclusion of Gerull et al.

#### Intraoperative complications

Five studies reported intraoperative complications (31–33, 36, 37). Events occurred in 1.46% (21/1,419) of RHHR patients and 3.06% (47/1,535) of LHHR patients. RHHR was associated with lower odds of intraoperative complications (OR 0.42, 95% CI 0.24–0.73; *p* = 0.002; I² = 42%) (Figure 8).

**Figure 8:**
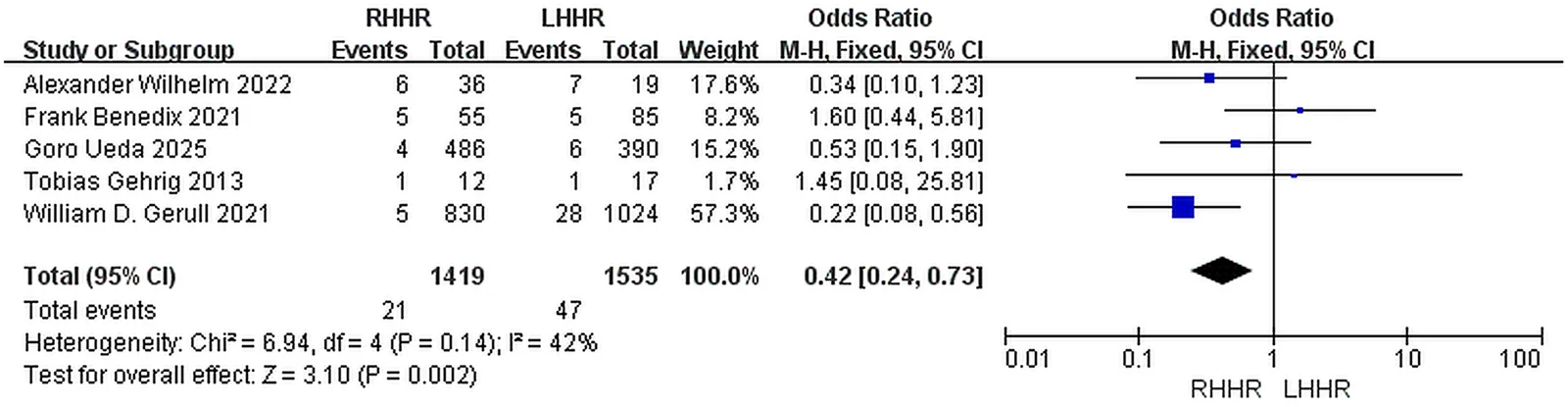
Forest plot of intraoperative complications.

#### Conversion to open surgery

Conversion to open surgery was reported inconsistently across the included studies (Table 3), with several large database studies providing no usable conversion data. Because reporting was sparse and definitions were not sufficiently uniform, conversion data were not pooled.

#### 30-day readmission

Five studies reported 30-day readmission (20, 23, 32, 33, 35). Readmissions occurred in 7.26% (4,442/61,191) of RHHR patients and 6.08% (28,169/463,184) of LHHR patients. RHHR was associated with higher odds of readmission (OR 1.23, 95% CI 1.19–1.27; *p* < 0.001; I² = 11%) (Figure 9).

**Figure 9:**
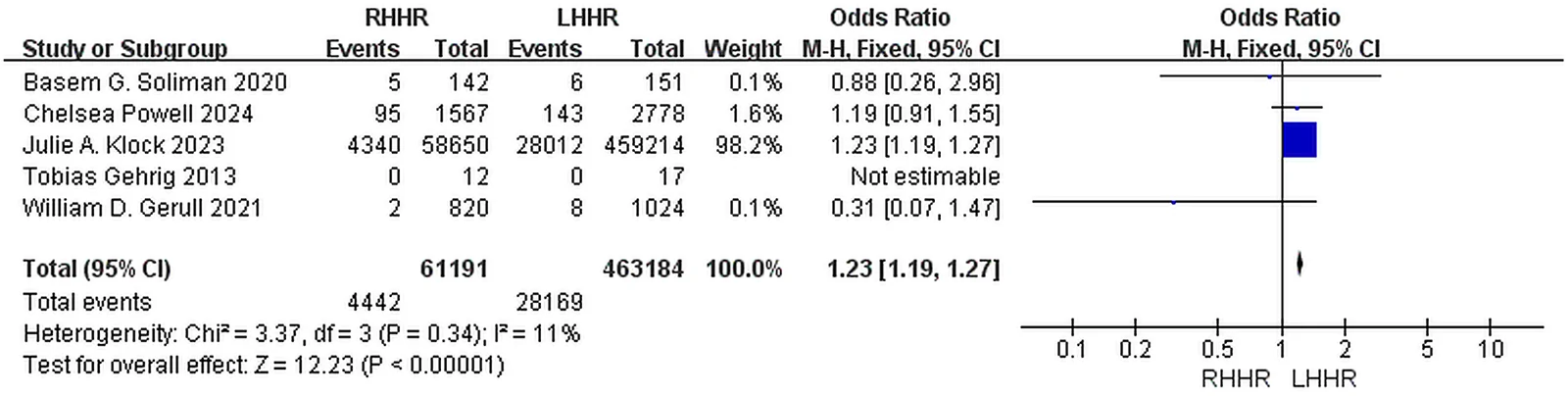
Forest plot of 30-day readmission.

#### Mortality

Nine studies reported mortality (20–23, 32–36). Mortality occurred in 0.39% (285/72,679) of RHHR patients and 0.31% (1,941/623,191) of LHHR patients. RHHR was associated with higher odds of mortality (OR 1.26, 95% CI 1.11–1.43; *p* < 0.001; I² = 48%) (Figure 10). The absolute difference was approximately 0.08 percentage points, emphasizing the importance of distinguishing statistical from clinical significance in large database analyses.

**Figure 10:**
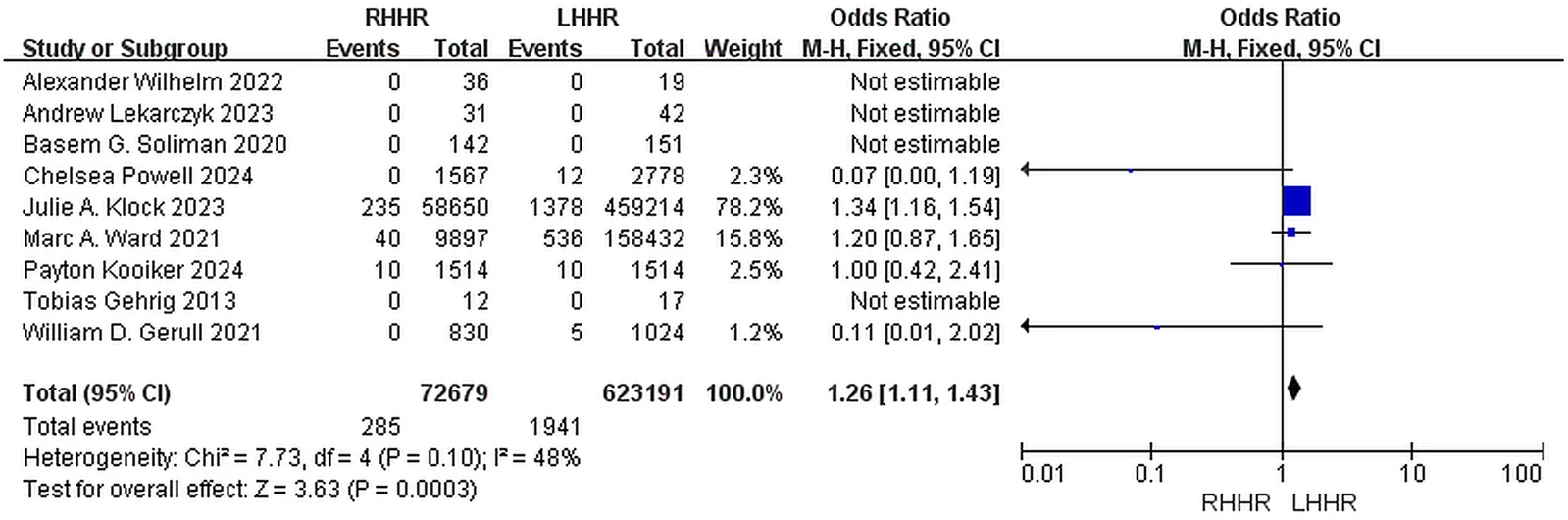
Forest plot of mortality.

#### Length of stay

Seven studies involving 6,293 patients reported length of stay (21, 31–35, 37). RHHR was associated with a modestly shorter hospital stay (MD −0.40 days, 95% CI −0.78–−0.02; *p* = 0.04; I² = 97%) (Figure 11). Subgroup analysis by region showed no significant difference in the Asian study (MD 0.00 days, 95% CI −0.22–0.22) or the US subgroup (MD −0.58 days, 95% CI −1.32–0.15), whereas the European subgroup favored RHHR (MD −0.39 days, 95% CI −0.62–−0.17). Between-subgroup heterogeneity was substantial (I² = 71.3%), suggesting that geographic setting may contribute to the overall inconsistency. Considerable heterogeneity also remained within the US subgroup (I² = 99%), indicating that region alone did not explain the variability.

**Figure 11:**
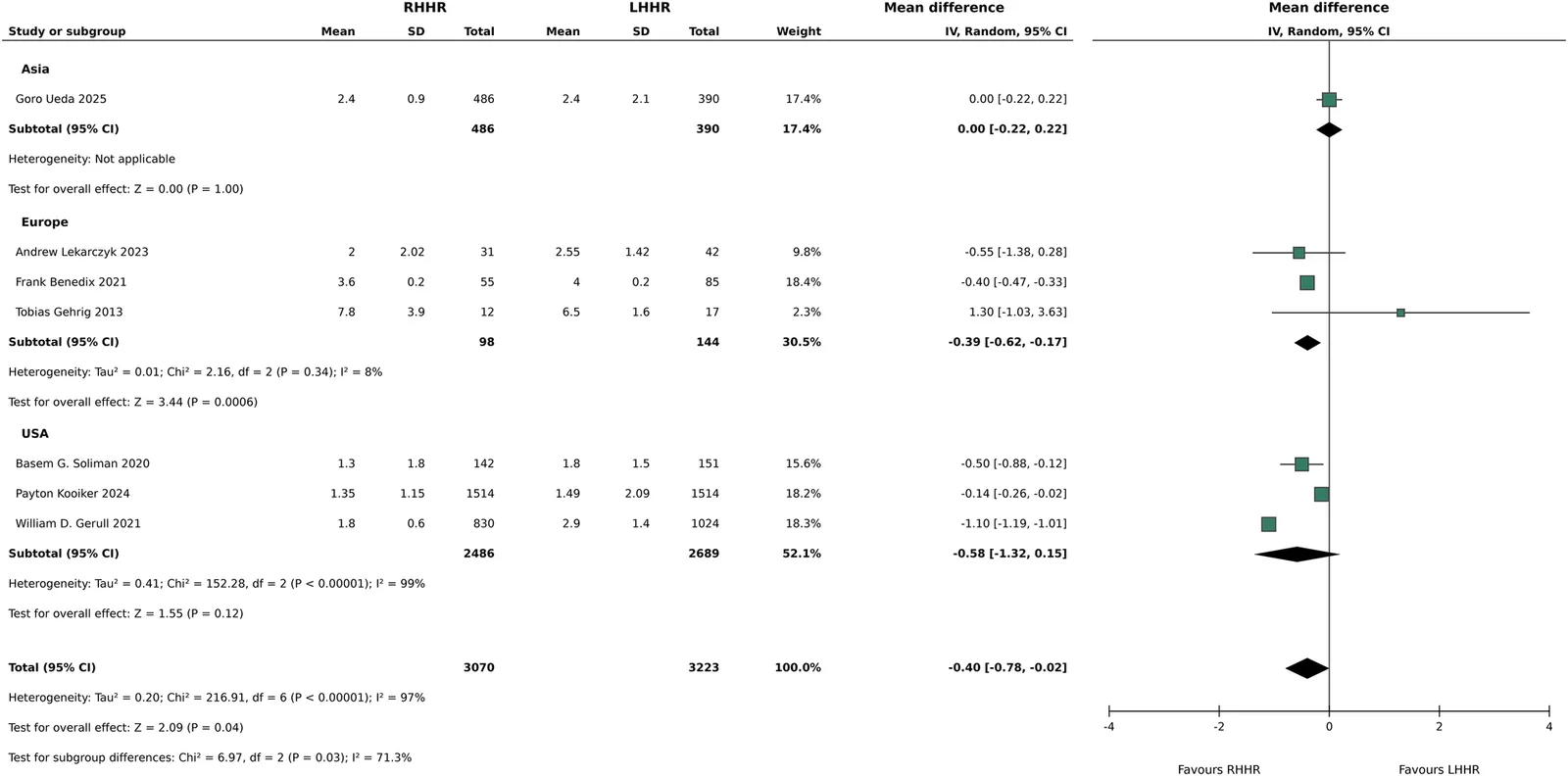
Forest plot of length of stay with geographic subgroup analysis.

### Supplementary sensitivity and sequential analyses

In leave-one-out analyses of overall postoperative complications, ORs ranged from 0.96 to 1.25 and I² remained between 81.0% and 87.0%, indicating that no single study accounted for the substantial heterogeneity (Supplementary Figure S1). For urological complications, ORs ranged from 0.99 to 1.44 and I² from 33.8% to 72.0%. Exclusion of Soliman et al. reduced I² to 33.8% and yielded a fixed-effect OR of 1.44 (95% CI 1.36–1.52); the confidence intervals for the other four leave-one-out analyses included 1 (Supplementary Figure S2).

For operative time, leave-one-out mean differences ranged from 7.73 to 22.95 min. Exclusion of Gerull et al. reduced I² to 39.6% and yielded the fixed-effect MD of 22.95 min (95% CI 16.71–29.19), consistent with the original exclusion analysis. All other leave-one-out analyses showed substantial heterogeneity and confidence intervals that included 0 (Supplementary Figure S3). Length-of-stay estimates remained negative, ranging from −0.49 to −0.25 days, although three of seven confidence intervals included 0; I² ranged from 79.9% to 97.7% (Supplementary Figure S4).

For overall postoperative complications, exclusion of studies with original NOS scores ≤6 yielded an OR of 1.27 (95% CI 1.11–1.46; I² = 86.2%). In contrast, exclusion of all four large database studies reduced I² to 43.2% and yielded a fixed-effect OR of 0.49 (95% CI 0.35–0.69), indicating sensitivity to study source (Supplementary Figure S5).

After exclusion of studies with NOS scores ≤6, two studies contributed to the operative-time analysis (fixed-effect MD 28.89 min, 95% CI 19.77–38.00; I² = 0%) and three to the length-of-stay analysis (MD −0.32 days, 95% CI −0.53–−0.10; I² = 86.0%). Detailed estimates, contributing studies, and exclusion scenarios in which no contributing study was removed are reported in Supplementary Tables S4–S5.

In the exploratory meta-regression, large database or registry studies were associated with higher estimated ORs for overall postoperative complications than other studies (log-OR coefficient 0.962, 95% CI 0.406–1.518; *p* = 0.0035; ratio of ORs 2.62, 95% CI 1.50–4.56; Supplementary Table S6).

The accumulated inverse-variance information was 159.77 units. For a target OR of 0.90, power calibration with nonbinding futility yielded a required maximum information level of 714.77 units, of which 22.4% had accumulated; no observed efficacy or futility boundary was crossed. For a target OR of 0.80, the required maximum was 188.35 units and the accumulated fraction was 84.8%. The first crossing of the upper efficacy boundary occurred after the study by Powell et al. was added (Z = 2.49; boundary = 2.28). After the subsequent addition of the Ueda study, Z was 1.66, below the efficacy boundary of 2.28 but above the positive futility boundary of 1.60; the later attenuation did not negate the earlier crossing. The repeated 95% CIs for the OR at the final observed assessment were 0.82–1.59 and 0.95–1.36 for the two scenarios, respectively. These are supplementary repeated intervals, not replacements for the primary confidence interval. The planning-only terminal point contained no additional observed data (Supplementary Table S7; Supplementary Figures S6,S7).

## Discussion

### Summary and comparison with previous evidence

In this meta-analysis, overall postoperative morbidity was not significantly different between robotic and laparoscopic hiatal hernia repair. RHHR was associated with fewer intraoperative complications and a modest reduction in length of stay, whereas higher odds of cardiovascular events, respiratory complications, 30-day readmission, and mortality were observed in the robotic group. No significant differences were identified for urological complications, dysphagia, or overall operative time. These findings should be interpreted as associations rather than causal effects because most of the evidence was observational and large administrative datasets contributed substantial statistical weight.

The magnitude and direction of several findings differ from prior meta-analyses. Ma et al. reported lower rates of postoperative complications and shorter length of stay with robotic repair in an earlier seven-study synthesis (14). A later meta-analysis of anti-reflux and hiatal hernia surgery found no significant differences in operative time, intraoperative complications, length of stay, readmission, overall complications, or mortality, while laparoscopic surgery was less costly (15). More recent systematic reviews likewise reported broadly comparable perioperative outcomes, although some identified longer operative times with robotic surgery or lower intraoperative complication rates (16–18). Differences among syntheses likely reflect evolving robotic platforms, the increasing use of large database studies, differing inclusion criteria, and heterogeneity in definitions of complications and operative time.

### Interpretation of organ-specific safety signals and case mix

A distinctive feature of the present analysis is the separation of organ-specific postoperative events from the aggregate complication endpoint. Cardiovascular event rates were 1.90% with RHHR and 1.87% with LHHR despite a statistically significant pooled OR of 1.24, illustrating how very large samples can yield statistical significance for small absolute differences. The difference in respiratory complication rates was larger (5.06% vs. 2.93%) and merits closer clinical attention. Nevertheless, residual confounding is a major concern. Robotic repair may be selected for larger, recurrent, type III/IV, or otherwise complex hernias, and administrative datasets may not fully adjust for these factors. In the extracted subtype data, type IV hernias appeared slightly more frequent among RHHR cases than LHHR cases (14.1% vs. 12.5%), consistent with a possible case-mix imbalance, although incomplete subtype reporting prevents firm inference. Consequently, the observed cardiopulmonary associations should not be attributed directly to the robotic platform.

### Operative time and intraoperative safety

Operative time was highly heterogeneous. Although the overall pooled estimate was not statistically significant, leave-one-out analysis identified Gerull et al. (33) as an important source of heterogeneity; after this study was excluded, RHHR was associated with a 22.95-minute longer operative time and heterogeneity decreased substantially. The duration of robotic surgery can be influenced by docking and setup, platform generation, team familiarity, the complexity of mediastinal dissection, and whether studies report total operating-room time or skin-to-skin time (38). It is therefore not justified to infer that longer robotic operative time caused the observed cardiopulmonary outcomes. Such a causal explanation would require patient-level adjustment and temporally appropriate mediation analyses.

Conversely, RHHR was associated with fewer intraoperative complications. Enhanced three-dimensional visualization, articulated instrumentation, tremor filtration, and the high spatial accuracy demonstrated in technical evaluations of da Vinci systems provide a plausible mechanism for improved precision during dissection and crural repair (39). However, only five studies contributed to this endpoint and the number of events was relatively small, so the apparent intraoperative advantage should be considered hypothesis-generating rather than definitive.

### Length of stay, regional variation, and institutional experience

RHHR was associated with an average reduction in length of stay of approximately 0.4 days, but heterogeneity was extreme. The regional subgroup analysis suggested shorter stays in European cohorts but no significant difference in the Asian or US subgroups. These differences may reflect institutional discharge practices, reimbursement systems, patient selection, surgeon experience, and differences in robotic platform generation rather than the surgical approach alone. The particularly high heterogeneity within the US subgroup supports this interpretation.

Institutional experience may also modify robotic outcomes. The included studies span more than a decade of robotic adoption, during which platform technology, docking efficiency, and team familiarity evolved. Prior observational experience and early randomized work in robotic foregut surgery suggest that technical adoption does not automatically translate into superior short-term clinical outcomes and that performance may change with experience (20, 40). Because surgeon case volume, center case volume, and learning-curve stage were not consistently reported, the present meta-analysis could not quantify this effect.

### Clinical implications

The pooled data do not support a simple conclusion that one minimally invasive approach is universally superior. The lower intraoperative complication rate and modestly shorter stay observed with RHHR may be relevant in selected complex repairs, whereas the higher pooled cardiopulmonary event, readmission, and mortality estimates emphasize the importance of preoperative risk stratification and careful case selection. In higher-risk patients, attention to cardiopulmonary optimization and postoperative surveillance remains appropriate regardless of operative platform. Because the observed differences are based mainly on non-randomized evidence, they should not be used alone to mandate or prohibit robotic repair.

### Limitations

This review has several limitations. First, the evidence base is predominantly observational, with substantial potential for selection bias, confounding by indication, and residual confounding. Second, national and multi-institutional databases contributed a large proportion of the total sample, and patient overlap across databases or study periods could not always be excluded. Third, definitions of overall and organ-specific complications varied among studies, particularly between clinical cohorts and administrative datasets. Fourth, detailed information on hernia size and type, redo status, comorbidity burden, fundoplication technique, mesh use, esophageal lengthening, robotic platform generation, surgeon and center volume, and learning-curve stage was inconsistently reported; therefore, efficacy and safety could not be reliably assessed by hiatal hernia subtype or procedural configuration. Fifth, several outcomes showed substantial statistical heterogeneity, particularly operative time and length of stay. Sixth, the synthesis primarily pooled unadjusted event data, whereas adjusted or propensity-matched effect estimates were not consistently available across studies. Seventh, conversion to open surgery was incompletely reported and could not be meaningfully pooled. Finally, the formal literature search was completed on 15 July 2025; studies published after this prespecified cutoff were outside the scope of the quantitative synthesis and may influence future updates. Future prospective studies should use standardized outcome definitions, detailed procedure- and subtype-stratified reporting, and adjusted analyses that account for case complexity and institutional experience.

The supplementary analyses indicate that some findings depend on the composition of the included studies. Heterogeneity in overall postoperative complications persisted after individual-study exclusions, and the estimates differed between NOS-restricted and database-restricted analyses. Operative-time estimates were particularly influenced by Gerull et al. Although all leave-one-out estimates favored a shorter hospital stay with RHHR, the confidence intervals did not consistently exclude zero. Because the model-selection rule depended on I², some changes reflected both study exclusion and a change in the fitted model.

The power-calibrated sequential assessment did not reach its planned information target in either scenario. For the smaller target effect, there was no observed efficacy crossing. For the larger effect, the upper efficacy boundary was crossed before the final study was added, after which the signal attenuated; the initial crossing remained part of the interpretation. These conditional retrospective findings do not establish equivalence, freedom from bias, or a definitive clinical advantage of either approach. The calculations depend on the chosen target effects, fixed heterogeneity, nominal within-study variances, and independence of the data sources. Further high-quality comparative evidence remains important, especially for smaller effects and standardized, reliably ascertained outcomes.

## Conclusions

Comparative evidence identified through 15 July 2025 suggests that RHHR and LHHR have broadly similar overall postoperative morbidity. RHHR may be associated with fewer intraoperative complications and a modestly shorter hospital stay, while higher pooled odds of cardiopulmonary events, readmission, and mortality require cautious interpretation because of confounding, database dominance, case-mix differences, and heterogeneous outcome definitions. The available data do not establish causal superiority or harm of either approach. Further prospective comparative studies, ideally including randomized trials where feasible, are needed, with standardized reporting of hernia subtype and operative technique and analyses using adjusted effect estimates to determine whether clinically meaningful safety differences exist between these two minimally invasive strategies.

## Data Availability

The original contributions presented in the study are included in the article/Supplementary Material, further inquiries can be directed to the corresponding author.

## References

[1] Stylopoulos N Rattner DW. The history of hiatal hernia surgery: from Bowditch to laparoscopy. Ann Surg. (2005) 241:185–93. 10.1097/01.sla.0000149430.83220.7f 15622007 PMC1356862

[2] Barrett NR. Hiatus hernia: a review of some controversial points. Br J Surg. (1954) 42:231–43. 10.1002/bjs.18004217303 13219304

[3] Wolf BS. Sliding hiatal hernia: the need for redefinition. Am J Roentgenol Radium Ther Nucl Med. (1973) 117:231–47. 10.2214/ajr.117.2.231 4734411

[4] Yu HX Han CS Xue JR Han ZF Xin H. Esophageal hiatal hernia: risk, diagnosis and management. Expert Rev Gastroenterol Hepatol. (2018) 12:319–29. 10.1080/17474124.2018.1441711 29451037

[5] Iwakiri K Fujiwara Y Manabe N Ihara E Kuribayashi S Akiyama J. Evidence-based clinical practice guidelines for gastroesophageal reflux disease 2021. J Gastroenterol. (2022) 57:267–85. 10.1007/s00535-022-01861-z 35226174 PMC8938399

[6] Dean C Etienne D Carpentier B Gielecki J Tubbs RS Loukas M. Hiatal hernias. Surg Radiol Anat. (2012) 34:291–9. 10.1007/s00276-011-0904-9 22105688

[7] Hosein S Carlson T Flores L Armijo PR Oleynikov D. Minimally invasive approach to hiatal hernia repair is superior to open, even in the emergent setting: a large national database analysis. Surg Endosc. (2021) 35:423–8. 10.1007/s00464-020-07404-y 32040632

[8] Toro JP Lin E Patel AD. Review of robotics in foregut and bariatric surgery. Surg Endosc. (2015) 29:1–8. 10.1007/s00464-014-3646-z 24972924

[9] Galvani CA Loebl H Osuchukwu O Samamé J Apel ME Ghaderi I. Robotic-assisted paraesophageal hernia repair: initial experience at a single institution. J Laparoendosc Adv Surg Tech A. (2016) 26:290–5. 10.1089/lap.2016.0096 27035739

[10] Brenkman HJ Parry K Van Hillegersberg R Ruurda JP. Robot-assisted laparoscopic hiatal hernia repair: promising anatomical and functional results. J Laparoendosc Adv Surg Tech A. (2016) 26:465–9. 10.1089/lap.2016.0065 27078499

[11] Telem DA. Is robotic surgery the future for abdominal wall hernia repair? Not so fast. Ann Surg. (2018) 267:218–9. 10.1097/SLA.0000000000002336 28632516

[12] Tsui C Klein R Garabrant M. Minimally invasive surgery: national trends in adoption and future directions for hospital strategy. Surg Endosc. (2013) 27:2253–7. 10.1007/s00464-013-2973-9 23660720

[13] Tonelli CM Baker MS Luchette FA Cohn T. Laparoscopic and robotic paraesophageal hernia repair in United States veterans: clinical outcomes and risk factors associated with reoperation recurrence. Am J Surg. (2023) 225:519–22. 10.1016/j.amjsurg.2022.12.013 36642563

[14] Ma L Luo H Kou S Gao Z Bai D Qin X. Robotic versus laparoscopic surgery for hiatal hernia repair: a systematic literature review and meta-analysis. J Robot Surg. (2023) 17:1879–90. 10.1007/s11701-023-01636-5 37247119

[15] Gonçalves-Costa D Barbosa JP Quesado R Lopes V Barbosa J. Robotic surgery versus laparoscopic surgery for anti-reflux and hiatal hernia surgery: a short-term outcomes and cost systematic literature review and meta-analysis. Langenbecks Arch Surg. (2024) 409:175. 10.1007/s00423-024-03368-y PMC11156741 38842610

[16] AlFataftah S Hammadeh BM Yaghi G AlFataftah S Al Shuqran B Abuhadba I. Safety and efficacy of robotic versus laparoscopic hiatal hernia repair: a systematic review and meta-analysis of perioperative outcomes. Hernia. (2025) 29:305. 10.1007/s10029-025-03484-8 41107615

[17] Mathys A Vanommeslaeghe H Van Nieuwenhove Y Van Daele E. A systematic review and meta-analysis: robotic versus laparoscopic hiatal hernia repair, is there a difference in Clavien Dindo score? Acta Chir Belg. (2026) 126:33–42. 10.1080/00015458.2025.2611452 41481122

[18] Xiao X Zhang C Li H Chen J Zhang J Yu W. Robotic-assisted versus laparoscopic esophageal hiatal hernia and anti-reflux surgery: a comprehensive systematic review and meta-analysis. Hernia. (2026) 30:273. 10.1007/s10029-026-03770-z 42397587

[19] Page MJ McKenzie JE Bossuyt PM Boutron I Hoffmann TC Mulrow CD. The PRISMA 2020 statement: an updated guideline for reporting systematic reviews. Br Med J. (2021) 372:n71. 10.1136/bmj.n71 PMC8005924 33782057

[20] Klock JA Walters RW Nandipati KC. Robotic hiatal hernia repair associated with higher morbidity and readmission rates compared to laparoscopic repair: 10-year analysis from the National Readmissions Database (NRD). J Gastrointest Surg. (2023) 27:489–97. 10.1007/s11605-022-05548-x 36508133

[21] Kooiker P Monnett S Thompson S Richmond B. Robotic-assisted versus laparoscopic repair of type II, III and IV hiatal hernias: a retrospective study comparing adverse outcomes. Laparosc Endosc Robot Surg. (2024) 7:11–5. 10.1016/j.lers.2023.12.004

[22] Ward MA Hasan SS Sanchez CE Whitfield EP Ogola GO Leeds SG. Complications following robotic hiatal hernia repair are higher compared to laparoscopy. J Gastrointest Surg. (2021) 25:3049–55. 10.1007/s11605-021-05005-1 33852128

[23] Powell C DeGregorio A Bews K Wigle D Habermann E. Perioperative outcomes of robotic versus laparoscopic paraesophageal hernia repair: a NSQIP analysis. Surg Endosc. (2024) 38:5851–7. 10.1007/s00464-024-11158-2 39160316 PMC13625184

[24] Dindo D Demartines N Clavien PA. Classification of surgical complications: a new proposal with evaluation in a cohort of 6336 patients and results of a survey. Ann Surg. (2004) 240:205–13. 10.1097/01.sla.0000133083.54934.ae 15273542 PMC1360123

[25] Stang A. Critical evaluation of the Newcastle-Ottawa scale for the assessment of the quality of nonrandomized studies in meta-analyses. Eur J Epidemiol. (2010) 25:603–5. 10.1007/s10654-010-9491-z 20652370

[26] Wells GA Shea B O’Connell D Peterson J Welch V Losos M. The Newcastle–Ottawa Scale (NOS) for assessing the quality of nonrandomised studies in meta-analyses. Ottawa Hospital Research Institute. Available online at: https://ohri.ca/en/who-we-are/core-facilities-and-platforms/ottawa-methods-centre/newcastle-ottawa-scale (Accessed September 24, 2026).

[27] Deeks JJ Higgins JPT Altman DG McKenzie JE Veroniki AA. Analysing data and undertaking meta-analyses. In: Higgins JPT Thomas J Chandler J Cumpston M Li T Page MJ. editors. Cochrane Handbook for Systematic Reviews of Interventions, Chapter 10. Cochrane. Available online at: https://www.cochrane.org/authors/handbooks-and-manuals/handbook/current/chapter-10 (Accessed October 8, 2026).

[28] Wetterslev J Jakobsen JC Gluud C. Trial sequential analysis in systematic reviews with meta-analysis. BMC Med Res Methodol. (2017) 17:39. 10.1186/s12874-017-0315-7 28264661 PMC5397700

[29] Lan KKG DeMets DL. Discrete sequential boundaries for clinical trials. Biometrika. (1983) 70:659–63. 10.1093/biomet/70.3.659

[30] Thorlund K Engstrøm J Wetterslev J Brok J Imberger G Gluud C. User Manual for Trial Sequential Analysis (TSA), 2nd Edn. Copenhagen: Copenhagen Trial Unit (2017). Available online at: https://ctu.dk/tools (Accessed October 8, 2026).

[31] Benedix F Adolf D Peglow S Gstettenbauer LM Croner R. Short-term outcome after robot-assisted hiatal hernia and anti-reflux surgery—is there a benefit for the patient? Langenbecks Arch Surg. (2021) 406:1387–95. 10.1007/s00423-020-02051-2 33409583

[32] Gehrig T Mehrabi A Fischer L Kenngott H Hinz U Gutt CN. Robotic-assisted paraesophageal hernia repair—a case-control study. Langenbecks Arch Surg. (2013) 398:691–6. 10.1007/s00423-012-0982-0 22846911

[33] Gerull WD Cho D Arefanian S Kushner BS Awad MM. Favorable peri-operative outcomes observed in paraesophageal hernia repair with robotic approach. Surg Endosc. (2021) 35:3085–9. 10.1007/s00464-020-07700-7 32556775

[34] Lekarczyk A Sinha H Dvir D Goyert J Airhart A Reddy RM. Similar hospital profits with robotic-assisted paraesophageal hiatal hernia repair, despite higher or supply costs. Surg Endosc. (2023) 37:3952–5. 10.1007/s00464-022-09513-2 35999312

[35] Soliman BG Nguyen DT Chan EY Chihara RK Meisenbach LM Graviss EA. Robot-assisted hiatal hernia repair demonstrates favorable short-term outcomes compared to laparoscopic hiatal hernia repair. Surg Endosc. (2020) 34:2495–502. 10.1007/s00464-019-07055-8 31385076

[36] Wilhelm A Nocera F Schneider R Koechlin L Daume DL Fourie L. Robot-assisted vs. Laparoscopic repair of complete upside-down stomach hiatal hernia (the RATHER-study): a prospective comparative single center study. Surg Endosc. (2022) 36:480–8. 10.1007/s00464-021-08307-2 33523279

[37] Ueda G Qaraqe T Han S Stiles E Sternback J Kuppusamy MK. Operative trends and clinical outcomes of open, laparoscopic and robotic approaches to hiatal and paraoesophageal hernias—a study of 1834 patients. J Robot Surg. (2025) 19:145. 10.1007/s11701-025-02299-0 40210798

[38] O'Connor SC Mallard M Desai SS Couto F Gottlieb M Ewing A. Robotic versus laparoscopic approach to hiatal hernia repair: results after 7 years of robotic experience. Am Surg. (2020) 86:1083–7. 10.1177/0003134820943547 32809844

[39] Ferguson JM Pitt B Kuntz A Granna J Kavoussi NL Nimmagadda N. Comparing the accuracy of the da Vinci Xi and da Vinci Si for image guidance and automation. Int J Med Robot. (2020) 16:e2149. 10.1002/rcs.2149 PMC9540612 32808429

[40] Müller-Stich BP Reiter MA Wente MN Bintintan VV Köninger J Büchler MW. Robot-assisted versus conventional laparoscopic fundoplication: short-term outcome of a pilot randomized controlled trial. Surg Endosc. (2007) 21:1800–5. 10.1007/s00464-007-9268-y 17353978

