# Supplementary material for: Perioperative safety profile of robotic versus laparoscopic hiatal hernia repair: a systematic review and meta-analysis
Source: Front Surg. 2026 Oct 21;13:1993235. doi: 10.3389/fsurg.2026.1993235 (PMC13731218; doi:10.3389/fsurg.2026.1993235)
Supplement: Supplementary file 1 [file Table1.docx]

# Supplementary Table S1. GRADE summary of findings

| **Outcome** | **Studies** | **Participants** | **Pooled effect (95% CI)** | **I²** | **Certainty of evidence (GRADE)** |
| --- | --- | --- | --- | --- | --- |
| Overall postoperative complications | 11 | 696,886 | OR 1.14 (0.98–1.33) | 86% | Very low (⊕◯◯◯)ᵇᶜᵈ |
| Cardiovascular events | 11 | 696,886 | OR 1.24 (1.17–1.31) | 15% | Very low (⊕◯◯◯)ᵇ |
| Respiratory complications | 9 | 694,156 | OR 1.65 (1.59–1.71) | 48% | Very low (⊕◯◯◯)ᵇ |
| Urological complications | 5 | 525,585 | OR 1.39 (0.88–2.17) | 63% | Very low (⊕◯◯◯)ᶜᵈ |
| Postoperative dysphagia | 3 | 1,027 | OR 1.23 (0.81–1.88) | 0% | Very low (⊕◯◯◯)ᵇᵈ |
| Operative time | 6 | 3,255 | MD 12.24 min (−7.10 to 31.58) | 93% | Very low (⊕◯◯◯)ᵇᵈᵉ |
| Intraoperative complications | 5 | 2,954 | OR 0.42 (0.24–0.73) | 42% | Very low (⊕◯◯◯)ᵇᵈ |
| 30-day readmission | 5 | 524,375 | OR 1.23 (1.19–1.27) | 11% | Low (⊕⊕◯◯) |
| Mortality | 9 | 695,870 | OR 1.26 (1.11–1.43) | 48% | Low (⊕⊕◯◯) |
| Length of stay | 7 | 6,293 | MD −0.40 d (−0.78 to −0.02) | 97% | Very low (⊕◯◯◯)ᵇᵈᵉ |

Abbreviations: CI, confidence interval; GRADE, Grading of Recommendations Assessment, Development and Evaluation; MD, mean difference; OR, odds ratio.

Note a. All included evidence was observational comparative evidence and therefore started at low certainty.

Note b. Downgraded for serious risk of bias because residual confounding/confounding by indication and limited between-group comparability remained plausible in contributing cohorts.

Note c. Downgraded for serious inconsistency because of substantial between-study heterogeneity.

Note d. Downgraded for serious imprecision because the 95% CI crossed the line of no effect and/or included clinically important benefit and harm, or because event counts were sparse.

Note e. Downgraded for very serious inconsistency. For operative time, heterogeneity was extreme (I²=93%) and sensitivity analysis materially changed the pooled conclusion; for length of stay, heterogeneity was extreme (I²=97%) with geographic subgroup differences.

Publication bias: Inability to perform a reliable formal assessment because of a small number of studies was not treated as an automatic reason for downgrading.

Counts and confidence intervals retain the reporting and sampling-design limitations described in the main manuscript and Tables S2 and S7.

# Supplementary Table S2. Study-specific outcome definitions

The definitions below are transcribed or paraphrased from the previously completed source review. PDF page references refer to the supplied original articles. Differences between source definitions and archived extraction are retained explicitly, rather than silently reconciled.

| **Study (main reference)** | **Definition and observation window** | **Important distinctions** | **Source location** |
| --- | --- | --- | --- |
| Benedix 2021 (31) | Any deviation from postoperative course, including Clavien–Dindo I–V. At least three-month follow-up is explicitly stated; procedure-related readmissions are collected within that period. | Operative time, intraoperative problems, hospital LOS and postoperative complications recorded from the institutional database. Three-month readmission is distinct from 30-day readmission unless separately reported. | Frank Benedix 2021.pdf, pp.2–3, Data collection / Statistical analysis. |
| Gehrig 2013 (32) | Postoperative complications, LOS and mortality registered at the end of the hospital stay; Clavien classification. | Intraoperative events recorded at operation end. Robotic operative time includes docking but excludes setup/demounting; laparoscopy is incision-to-closure. | Tobias Gehrig 2013.pdf, p.2, Surgical outcome; pp.3–4, outcome tables. |
| Gerull 2021 (33) | Postoperative complications and reoperation within 30 days; mortality described as 30-day in-hospital mortality. | Skin-incision-to-closure operative time; intraoperative injury and conversion separately reported. LOS units and source Table 2 must be used rather than an unverified workbook version. | William D. Gerull 2021.pdf, pp.2–3, Methods / Table 2. |
| Klock 2023 (20) | ICD-coded postprocedural events during the index hospitalization. All-cause readmissions measured at 30 and 90 days; death is in-hospital. | NRD survey-weighted national hospitalization estimates; specific complication codes described with Supplemental Table S1, not included as a separate file in this archive. | Julie A. Klock 2023.pdf, pp.2–4, Methods / Table 2; separate code supplement pending. |

# Supplementary Table S2. Study-specific outcome definitions (continued)

| **Study (main reference)** | **Definition and observation window** | **Important distinctions** | **Source location** |
| --- | --- | --- | --- |
| Kooiker 2024 (21) | Outcome query window is 30 days after index repair. Table 3 reports separate adverse events, critical care, ED visits and mortality. | LOS reported in hours (32.4±27.5 vs 35.7±50.1). No explicitly de-duplicated at-least-one-complication endpoint is shown in Table 3; summing rows is not a patient-level composite. | Payton Kooiker 2024.pdf, p.2 §2.3; p.4 Table 3. |
| Lekarczyk 2023 (34) | Table 2 gives patients with/without complications, individual event types and mortality after 30 days. | Includes pneumonia, procedure-related readmission, unanticipated procedure, chylothorax and treated arrhythmia. LOS and operative time are mean±SD; the separate mortality label alone does not define every event’s window. | Andrew Lekarczyk 2023.pdf, pp.2–3, Methods / Table 2. |
| Soliman 2020 (35) | Postoperative morbidity recorded from a prospectively collected institutional STS database; outcomes and analyses reported in Tables 2–4. | Original 142/151 cohort and separate age/ASA-matched 141/141 cohort are both described. Cohort versions and their outcome definitions are distinct. | Basem G. Soliman 2020.pdf, pp.2–4, Methods / Tables 2–4; matched supplements cited. |
| Wilhelm 2022 (36) | Complications are deviations during surgery or postoperative course; all events counted; primary morbidity/mortality window is 30 days. Major morbidity is Clavien–Dindo ≥IIIb. | Multiple events per patient are explicitly possible. Telephone symptoms/QoL assessed at 8–53 months are different from 30-day complications. A dichotomous composite requires affected-patient counts rather than the sum of events. | Alexander Wilhelm 2022.pdf, pp.2–3, Follow-up / Study endpoints; Tables 2–3. |

# Supplementary Table S2. Study-specific outcome definitions (continued)

| **Study (main reference)** | **Definition and observation window** | **Important distinctions** | **Source location** |
| --- | --- | --- | --- |
| Ward 2021 (22) | NIS-coded complications, in-hospital mortality and hospital LOS. | Codes and event definitions must follow the original report. Design uses weighting and hospital-level random intercepts. NIS discharge records do not establish 30-day post-discharge ascertainment. | Marc A. Ward 2021.pdf, pp.2–4, Methods / Tables. |
| Powell 2024 (23) | NSQIP 30-day major composite includes listed major events, reoperation and death; minor composite includes superficial SSI and UTI. | Readmission modeled using days at risk; LOS categorized as same day/overnight/≥2 nights. Sums of individual components do not necessarily represent unique patients with any morbidity. | Chelsea Powell2024.pdf, p.2 Outcomes / Statistical analysis; pp.3–5 outcome tables. |
| Ueda 2025 (37) | Operative and postoperative outcomes are in Tables 3–5; symptom prevalence and improvement are separately reported in Table 2. | Postoperative heartburn is not a cardiovascular complication. Symptoms use 452/380 evaluable robotic/laparoscopic patients, not the full 486/390 cohort. Cohort is Virginia Mason, Seattle, USA. | Goro Ueda2025.pdf, pp.2–3 Methods; p.5 Table 2; pp.6–7 Tables 3–5; p.10 affiliation 1. |

# Supplementary Table S3. Original NOS ratings and scoring guidance

The original cohort-NOS ratings in main Table 4 are retained. The item-level guidance was expanded during revision to make the assessment transparent; the source checkpoints below do not imply that a new independent reassessment or a new consensus score has been completed (25, 26).

| **Study** | **S1** | **S2** | **S3** | **S4** | **C /2** | **O1** | **O2** | **O3** | **Total /9** |
| --- | --- | --- | --- | --- | --- | --- | --- | --- | --- |
| Benedix 2021 (31) | ★ | ★ | ★ | ★ | ★★ | ★ | ★ | - | 8 |
| Gehrig 2013 (32) | ★ | ★ | ★ | ★ | - | ★ | ★ | - | 6 |
| Gerull 2021 (33) | ★ | ★ | ★ | ★ | - | ★ | ★ | - | 6 |
| Klock 2023 (20) | ★ | ★ | ★ | ★ | ★★ | ★ | ★ | - | 8 |
| Kooiker 2024 (21) | ★ | ★ | ★ | ★ | ★★ | ★ | ★ | - | 8 |
| Lekarczyk 2023 (34) | ★ | ★ | ★ | ★ | - | ★ | ★ | - | 6 |
| Soliman 2020 (35) | ★ | ★ | ★ | ★ | ★ | ★ | ★ | - | 7 |
| Wilhelm 2022 (36) | ★ | ★ | ★ | ★ | - | ★ | ★ | ★ | 7 |
| Ward 2021 (22) | ★ | ★ | ★ | ★ | ★★ | ★ | ★ | - | 8 |
| Powell 2024 (23) | ★ | ★ | ★ | ★ | ★★ | ★ | ★ | - | 8 |
| Ueda 2025 (37) | ★ | ★ | ★ | ★ | - | ★ | ★ | - | 6 |

S1–S4: selection; C: comparability; O1–O3: outcome assessment. Each item is worth at most one star except comparability (two). A dash denotes a retained non-award; it does not by itself distinguish a methodological defect from insufficient reporting.

The NOS≤6 sensitivity subset consists of Gehrig 2013, Gerull 2021, Lekarczyk 2023 and Ueda 2025. The cut-off is a review-specific sensitivity threshold, not a universal NOS risk category. Earlier provisional 5–9-point ratings are not used in this supplement.

# Table S3 (continued). Operational guidance

| **NOS item** | **Scoring guidance (26)** |
| --- | --- |
| S1 · Representativeness | A truly or reasonably representative RHHR cohort from a defined source population; record consecutive enrollment or sampling, setting, study period and selection restrictions. A large database alone does not establish representativeness. |
| S2 · Comparator source | An LHHR group drawn from the same source population with comparable eligibility. Institution, period and surgeon differences are recorded separately as possible confounding. |
| S3 · Exposure ascertainment | Secure surgical or clinical records, appropriately documented procedure coding, or adequate structured ascertainment of the surgical approach and conversions. |
| S4 · Outcome absent at baseline | Evidence that the assessed event was absent at entry or explicitly defined as incident after the operation. Postoperative documentation alone does not distinguish new events from persistent symptoms. |
| C · Comparability | One star for control of the review-designated most important confounder and a second for another relevant factor, through matching or adjustment. Nonsignificant baseline comparisons are insufficient. The actual variables and the data version pooled must be recorded; unmatched counts do not inherit adjustment from another model. |
| O1 · Outcome ascertainment | Independent assessment or secure clinical records/record linkage. Lack of blinding does not automatically invalidate reliably recorded objective outcomes. |
| O2 · Duration of follow-up | Assess against the actual endpoint: operative period, discharge/death for in-hospital outcomes, or an adequate 30-day window for 30-day outcomes. These windows are not interchangeable. |
| O3 · Completeness of follow-up | All participants accounted for or documented losses unlikely to bias the comparison. Record missingness and reasons by group. A follow-up schedule or large sample alone does not establish completeness. |

# Table S3 (continued). Source checkpoints

| **Study / retained total** | **Source location** | **Evidence checkpoint or unresolved distinction** |
| --- | --- | --- |
| Benedix · 8 (31) | pp.2–3, Data collection / Statistical analysis | The source states all patients were followed for at least three months; retained O3=0 requires an explicit explanation tied to the assessed endpoint. Original 55/85 and matched 48/48 are distinct. |
| Gehrig · 6 (32) | p.2, Surgical outcome / Statistical analysis | Record objective in-hospital assessment and any completeness statement. A descriptive “well matched” claim is not a matching procedure. |
| Gerull · 6 (33) | pp.2–3, Methods / Table 2 | Record comparator source, surgeon differences and short-term completion. Chi-square/t tests alone do not establish confounder control. |
| Klock · 8 (20) | pp.2–4, Methods / Tables 1–2 | Document actual age/clinical adjustments and outcome codes. Weighted national totals and design-adjusted precision are separate from NOS stars. |
| Kooiker · 8 (21) | p.2 §§2.2–2.3; p.4 Table 3 | Match variables are documented; retained S4 and O3 require endpoint-specific checks. A 30-day query does not prove absence at baseline or complete observation. |
| Lekarczyk · 6 (34) | pp.2–3, Methods / Tables 1–2 | Confirm the principal morbidity observation window supporting O2=1; a separate mortality label does not define every complication window. |

# Table S3 (continued). Source checkpoints (continued)

These checkpoints preserve source-supported information and discrepancies identified in the original-file review. Supporting evidence and unresolved rating distinctions are reported separately. Ratings remain those in Table 4.

| **Study / retained total** | **Source location** | **Evidence checkpoint or unresolved distinction** |
| --- | --- | --- |
| Soliman · 7 (35) | pp.2–4, Methods / Tables 2–4 | Source describes age/ASA matching; retained C=1 needs a relevant-model/data-version explanation. 142/151 differs from matched 141/141. |
| Wilhelm · 7 (36) | pp.2–3, Follow-up / Study endpoints | Retained O3=1 needs short-term completeness evidence, not only the later 8–53-month symptom/QoL follow-up. |
| Ward · 8 (22) | pp.2–4, Methods / Tables | Confirm in-hospital event coding, adjusted factors and completeness; do not label NIS capture as post-discharge 30-day follow-up. |
| Powell · 8 (23) | pp.2–5, Methods / outcome tables | C pertains to the relevant adjusted outcome model. Sparse-event mortality was not adjusted in the same way. |
| Ueda · 6 (37) | pp.2–3,5–7,10 | Record surgical versus symptom endpoints and era differences. The retained score is not validation of the legacy endpoint/geography mapping. |

# Supplementary Table S4. Sensitivity-analysis scope and summary

Additional analyses were limited to outcomes with I² >50% in the original main analysis. Eligibility was determined before exclusions; results were retained even when reduced-data I² fell to ≤50%. Other outcomes were not subjected to additional sensitivity analysis in this revision. These are post hoc supplementary diagnostics, not replacement primary analyses (27).

Each reduced dataset followed the submitted model-selection rule: DerSimonian–Laird random effects for I² >50%, otherwise Mantel–Haenszel fixed effect for binary outcomes or inverse-variance fixed effect for continuous outcomes. Thus, the Gerull-excluded operative-time result retains the original fixed-effect model. Single-zero binary studies received a 0.5 correction in all four cells; double-zero studies did not estimate a relative effect. No multiple-testing adjustment was applied.

| **Outcome** | **Original I²** | **LOO runs** | **Estimate range** | **Reduced I² range** | **CI spans null** |
| --- | --- | --- | --- | --- | --- |
| Overall postoperative complications | 86% | 11 | 0.96 to 1.25 | 81.0–87.0% | 9/11 |
| Urological complications | 63% | 5 | 0.99 to 1.44 | 33.8–72.0% | 4/5 |
| Operative time (minutes) | 93% | 6 | 7.73 to 22.95 | 39.6–94.6% | 5/6 |
| Length of stay (days) | 97% | 7 | -0.49 to -0.25 | 79.9–97.7% | 3/7 |

LOO, leave one out; CI, confidence interval. The null is OR=1 for complications and MD=0 for continuous outcomes. Counts of intervals spanning the null are descriptive, not a vote-counting test.

# Table S4 (continued). Original-NOS and source restrictions

## Overall postoperative complications

| **Restriction** | **Studies** | **Model** | **Estimate [95% CI]** | **I²** |
| --- | --- | --- | --- | --- |
| Exclude NOS ≤6 | 7 | Random effects | 1.27 [1.11, 1.46] | 86.2% |
| Omit Klock / NRD | 10 | Random effects | 0.96 [0.75, 1.24] | 81.3% |
| Omit Ward / NIS | 10 | Random effects | 1.00 [0.78, 1.28] | 82.9% |
| Omit NRD and NIS | 9 | Random effects | 0.82 [0.54, 1.24] | 83.3% |
| Exclude all large databases | 7 | Fixed effect (MH) | 0.49 [0.35, 0.69] | 43.2% |

## Urological complications

| **Restriction** | **Studies** | **Model** | **Estimate [95% CI]** | **I²** |
| --- | --- | --- | --- | --- |
| Omit Klock / NRD | 4 | Random effects | 1.09 [0.39, 3.05] | 68.3% |
| Omit NRD and NIS | 4 | Random effects | 1.09 [0.39, 3.05] | 68.3% |
| Exclude all large databases | 2 | Fixed effect (MH) | 0.42 [0.15, 1.18] | 41.7% |

Original NOS≤6 excludes no contributing urological study. Omitting NRD and NIS gives the same data as omitting NRD because no NIS study contributed to this endpoint.

## Operative time (minutes)

| **Restriction** | **Studies** | **Model** | **Estimate [95% CI]** | **I²** |
| --- | --- | --- | --- | --- |
| Exclude NOS ≤6 | 2 | Fixed effect (IV) | 28.89 [19.77, 38.00] | 0.0% |

## Length of stay (days)

| **Restriction** | **Studies** | **Model** | **Estimate [95% CI]** | **I²** |
| --- | --- | --- | --- | --- |
| Exclude NOS ≤6 | 3 | Random effects | -0.32 [-0.53, -0.10] | 86.0% |
| Exclude all large databases | 6 | Random effects | -0.46 [-0.90, -0.01] | 97.1% |

# Table S4 (continued). Restrictions that were not applicable

| **Outcome** | **Restriction** | **Reason** |
| --- | --- | --- |
| Urological complications | Exclude NOS ≤6 | No contributing study was omitted. |
| Urological complications | Omit Ward / NIS | No contributing study was omitted. |
| Operative time (minutes) | Omit Klock / NRD | No contributing study was omitted. |
| Operative time (minutes) | Omit Ward / NIS | No contributing study was omitted. |
| Operative time (minutes) | Omit NRD and NIS | No contributing study was omitted. |
| Operative time (minutes) | Exclude all large databases | No contributing study was omitted. |
| Length of stay (days) | Omit Klock / NRD | No contributing study was omitted. |
| Length of stay (days) | Omit Ward / NIS | No contributing study was omitted. |
| Length of stay (days) | Omit NRD and NIS | No contributing study was omitted. |

Source-restriction analyses assess dependence on the included studies; they do not demonstrate patient-level deduplication. Known source-definition, nominal variance and sampling-design limitations are preserved in the analysis inputs and discussed in the manuscript.

# Supplementary Table S5. Leave-one-out results

## Overall postoperative complications

| **Omitted study** | **Studies left** | **Model** | **Estimate [95% CI]** | **I²** | **p** |
| --- | --- | --- | --- | --- | --- |
| Gehrig 2013 (32) | 10 | Random effects | 1.14 [0.98, 1.33] | 86.9% | 0.098 |
| Soliman 2020 (35) | 10 | Random effects | 1.21 [1.05, 1.40] | 83.3% | 0.007 |
| Benedix 2021 (31) | 10 | Random effects | 1.15 [0.98, 1.34] | 86.8% | 0.087 |
| Gerull 2021 (33) | 10 | Random effects | 1.16 [1.00, 1.36] | 86.2% | 0.055 |
| Ward 2021 (22) | 10 | Random effects | 1.00 [0.78, 1.28] | 82.9% | 0.984 |
| Wilhelm 2022 (36) | 10 | Random effects | 1.13 [0.97, 1.32] | 86.9% | 0.129 |
| Klock 2023 (20) | 10 | Random effects | 0.96 [0.75, 1.24] | 81.3% | 0.761 |
| Lekarczyk 2023 (34) | 10 | Random effects | 1.14 [0.97, 1.33] | 87.0% | 0.113 |
| Kooiker 2024 (21) | 10 | Random effects | 1.05 [0.88, 1.25] | 86.1% | 0.622 |
| Powell 2024 (23) | 10 | Random effects | 1.13 [0.95, 1.35] | 86.1% | 0.158 |
| Ueda 2025 (37) | 10 | Random effects | 1.25 [1.09, 1.43] | 81.0% | 0.001 |

OR compares RHHR with LHHR. MD is RHHR minus LHHR, in minutes or days as stated. Each row describes the remaining studies after the named study is omitted; it is not that study’s individual effect.

# Supplementary Table S5. Leave-one-out results (continued)

## Urological complications

| **Omitted study** | **Studies left** | **Model** | **Estimate [95% CI]** | **I²** | **p** |
| --- | --- | --- | --- | --- | --- |
| Soliman 2020 (35) | 4 | Fixed effect (MH) | 1.44 [1.36, 1.52] | 33.8% | <0.001 |
| Wilhelm 2022 (36) | 4 | Random effects | 1.36 [0.83, 2.21] | 72.0% | 0.220 |
| Klock 2023 (20) | 4 | Random effects | 1.09 [0.39, 3.05] | 68.3% | 0.868 |
| Kooiker 2024 (21) | 4 | Random effects | 0.99 [0.46, 2.12] | 52.0% | 0.980 |
| Powell 2024 (23) | 4 | Random effects | 1.38 [0.83, 2.32] | 71.8% | 0.218 |

OR compares RHHR with LHHR. MD is RHHR minus LHHR, in minutes or days as stated. Each row describes the remaining studies after the named study is omitted; it is not that study’s individual effect.

# Supplementary Table S5. Leave-one-out results (continued)

## Operative time (minutes)

| **Omitted study** | **Studies left** | **Model** | **Estimate [95% CI]** | **I²** | **p** |
| --- | --- | --- | --- | --- | --- |
| Gehrig 2013 (32) | 5 | Random effects | 13.59 [-7.95, 35.12] | 94.6% | 0.216 |
| Soliman 2020 (35) | 5 | Random effects | 7.73 [-12.32, 27.79] | 92.0% | 0.450 |
| Benedix 2021 (31) | 5 | Random effects | 9.69 [-12.48, 31.85] | 93.8% | 0.392 |
| Gerull 2021 (33) | 5 | Fixed effect (IV) | 22.95 [16.71, 29.19] | 39.6% | <0.001 |
| Lekarczyk 2023 (34) | 5 | Random effects | 13.92 [-7.31, 35.15] | 94.6% | 0.199 |
| Ueda 2025 (37) | 5 | Random effects | 10.20 [-13.41, 33.81] | 93.1% | 0.397 |

OR compares RHHR with LHHR. MD is RHHR minus LHHR, in minutes or days as stated. Each row describes the remaining studies after the named study is omitted; it is not that study’s individual effect.

Excluding Gerull retains fixed-effect MD 22.95 minutes [16.71, 29.19], I²=39.6%, matching the original exclusion analysis.

# Supplementary Table S5. Leave-one-out results (continued)

## Length of stay (days)

| **Omitted study** | **Studies left** | **Model** | **Estimate [95% CI]** | **I²** | **p** |
| --- | --- | --- | --- | --- | --- |
| Gehrig 2013 (32) | 6 | Random effects | -0.44 [-0.82, -0.06] | 97.7% | 0.023 |
| Soliman 2020 (35) | 6 | Random effects | -0.38 [-0.80, 0.04] | 97.7% | 0.073 |
| Benedix 2021 (31) | 6 | Random effects | -0.37 [-0.93, 0.19] | 97.4% | 0.190 |
| Gerull 2021 (33) | 6 | Random effects | -0.25 [-0.45, -0.05] | 79.9% | 0.016 |
| Lekarczyk 2023 (34) | 6 | Random effects | -0.39 [-0.79, 0.01] | 97.7% | 0.058 |
| Kooiker 2024 (21) | 6 | Random effects | -0.46 [-0.90, -0.01] | 97.1% | 0.043 |
| Ueda 2025 (37) | 6 | Random effects | -0.49 [-0.90, -0.07] | 97.4% | 0.021 |

OR compares RHHR with LHHR. MD is RHHR minus LHHR, in minutes or days as stated. Each row describes the remaining studies after the named study is omitted; it is not that study’s individual effect.

# Supplementary Table S6. Exploratory study-source meta-regression

| **Field** | **Result** |
| --- | --- |
| Outcome | Overall postoperative complications |
| Moderator | Large database/registry study versus other source |
| Studies | 11: four database/registry and seven other studies |
| Model | REML residual heterogeneity; modified Hartung–Knapp covariance adjustment |
| Log-OR coefficient | 0.9617 [0.4060, 1.5175] |
| Ratio of ORs | 2.616 [1.501, 4.561] |
| p | 0.003540 |
| Residual τ² | 0.018517 |
| Degrees of freedom | 9 |

This previously computed univariable analysis is retained without refitting. It is a post hoc study-level association, not a causal patient-level effect. No meta-regression was conducted for the other three high-heterogeneity endpoints, each supported by only five to seven studies (27).

The result is conditional on the archived outcome definitions and nominal variances. Database omission and a source moderator cannot establish that underlying patients are independent or resolve outcome-counting issues.

# Supplementary Table S7. Power-calibrated sequential information assessment

A post hoc information-based trial sequential assessment was performed for overall postoperative complications only (28–30). Studies were ordered by publication year and then by the first author’s surname within each year. Two-sided Lan–DeMets/O’Brien–Fleming efficacy boundaries were calculated using an overall alpha of 0.05. Target ORs of 0.90 and 0.80 were examined as illustrative assumptions, rather than established minimum clinically important differences. The first scenario served as the principal information assessment, and the second assessed dependence on the assumed effect size; the scenarios were not interpreted as independent confirmatory tests. The alternative effects were symmetric on the log-OR scale. Study-level log ORs and nominal variances were taken from the existing supplementary analysis data. Statistical information was calculated as the sum of inverse-variance weights, with between-study variance fixed at the previously estimated value of 0.026451; the primary analyses were not replaced.

Nonbinding symmetric futility boundaries were added using O’Brien–Fleming-type beta spending. The required maximum information level was determined numerically to obtain an 80% probability of crossing an efficacy boundary in the correct direction under each target effect when the specified stopping rules were followed. Efficacy boundaries were calculated without assuming futility stopping, preserving nominal type-I error control if futility was ignored. Calibration used the observed information times and one planning-only terminal assessment at the required maximum information; this terminal assessment included no additional observed study or outcome. Repeated 95% confidence intervals were obtained by applying the stage-specific efficacy boundary to the cumulative standard error. Numerical integration was checked at two quadrature resolutions and against independent Gaussian-increment simulations of 500,000 sequences per scenario. The algorithms, assumptions, parameters, and numerical checks are provided with Supplementary Table S7.

The assessment was implemented in Python with NumPy and SciPy. Calibration and coverage are conditional on a model with independent Gaussian score increments and fixed nuisance parameters; they do not establish prospective power for this retrospective dataset. Survey-weighted national counts, potential population overlap, and unresolved composite-outcome counting limit the clinical interpretation. The first efficacy-boundary crossing was recorded; subsequent observations were displayed retrospectively and did not negate the initial crossing. Entering a futility region would not establish equivalence, and neither a boundary crossing nor the attainment of an information target removes systematic bias.

# Table S7 (continued). Summary of the two scenarios

| **Metric** | **Target OR 0.90** | **Target OR 0.80** |
| --- | --- | --- |
| Accumulated information | 159.77 | 159.77 |
| Fixed-design planning information | 707.05 | 157.63 |
| Power-calibrated maximum information | 714.77 | 188.35 |
| Accumulated fraction | 22.4% | 84.8% |
| First observed efficacy crossing | None | Powell 2024 (upper) |
| Observed futility entry | None | None |
| Last observed Z / efficacy boundary | 1.66 / 4.19 | 1.66 / 2.28 |
| Last observed repeated 95% OR interval | 0.82–1.59 | 0.95–1.36 |

Information is measured in inverse-log-OR-variance units, not patients. The 100% target is a planning-only assessment with no observed study or Z value. OR=0.80 is an assumption comparison, not an independently selected confirmatory test.

Neither observed sequence entered a futility region. Under OR=0.80, the first upper efficacy-boundary crossing occurred when Powell was added; the later attenuation after Ueda does not erase that crossing. Repeated intervals supplement but do not replace the main-analysis confidence interval.

# Table S7 (continued). Sequence for target OR 0.90

| **Study / look** | **Info.** | **Fraction** | **Z** | **Efficacy bound** | **Futility bound** | **Crossing** |
| --- | --- | --- | --- | --- | --- | --- |
| Gehrig 2013 (32) | 0.59 | 0.1% | -0.30 | No finite test | ±0.000 | — |
| Soliman 2020 (35) | 5.92 | 0.8% | -2.84 | No finite test | ±0.000 | — |
| Benedix 2021 (31) | 9.06 | 1.3% | -2.50 | No finite test | ±0.000 | — |
| Gerull 2021 (33) | 11.77 | 1.6% | -2.83 | No finite test | ±0.000 | — |
| Ward 2021 (22) | 48.28 | 6.8% | -0.38 | No finite test | ±0.000 | — |
| Wilhelm 2022 (36) | 50.44 | 7.1% | -0.14 | ±7.378 | ±0.000 | — |
| Klock 2023 (20) | 87.91 | 12.3% | 1.25 | ±5.589 | ±0.001 | — |
| Lekarczyk 2023 (34) | 89.61 | 12.5% | 1.30 | ±5.577 | ±0.000 | — |
| Kooiker 2024 (21) | 120.13 | 16.8% | 2.43 | ±4.782 | ±0.004 | — |
| Powell 2024 (23) | 149.79 | 21.0% | 2.49 | ±4.289 | ±0.010 | — |
| Ueda 2025 (37) | 159.77 | 22.4% | 1.66 | ±4.190 | ±0.005 | — |
| Planning-only target | 714.77 | 100.0% | Not observed | ±1.960 | ±1.960 | — |

Studies were ordered by year and first-author surname within each year, not by reconstructed exact online-publication dates. No finite test denotes negligible allocated alpha at an early look. Connecting lines in the plots guide the eye; tests refer to the listed information times.

# Table S7 (continued). Sequence for target OR 0.80

| **Study / look** | **Info.** | **Fraction** | **Z** | **Efficacy bound** | **Futility bound** | **Crossing** |
| --- | --- | --- | --- | --- | --- | --- |
| Gehrig 2013 (32) | 0.59 | 0.3% | -0.30 | No finite test | ±0.000 | — |
| Soliman 2020 (35) | 5.92 | 3.1% | -2.84 | No finite test | ±0.000 | — |
| Benedix 2021 (31) | 9.06 | 4.8% | -2.50 | No finite test | ±0.000 | — |
| Gerull 2021 (33) | 11.77 | 6.2% | -2.83 | No finite test | ±0.000 | — |
| Ward 2021 (22) | 48.28 | 25.6% | -0.38 | ±3.871 | ±0.047 | — |
| Wilhelm 2022 (36) | 50.44 | 26.8% | -0.14 | ±3.846 | ±0.010 | — |
| Klock 2023 (20) | 87.91 | 46.7% | 1.25 | ±2.874 | ±0.498 | — |
| Lekarczyk 2023 (34) | 89.61 | 47.6% | 1.30 | ±2.929 | ±0.425 | — |
| Kooiker 2024 (21) | 120.13 | 63.8% | 2.43 | ±2.499 | ±1.066 | — |
| Powell 2024 (23) | 149.79 | 79.5% | 2.49 | ±2.279 | ±1.521 | Efficacy |
| Ueda 2025 (37) | 159.77 | 84.8% | 1.66 | ±2.277 | ±1.600 | — |
| Planning-only target | 188.35 | 100.0% | Not observed | ±2.092 | ±2.092 | — |

Studies were ordered by year and first-author surname within each year, not by reconstructed exact online-publication dates. No finite test denotes negligible allocated alpha at an early look. Connecting lines in the plots guide the eye; tests refer to the listed information times.

# Table S7 (continued). Numerical implementation and archived checks

For study i, yᵢ = log(aᵢdᵢ/bᵢcᵢ) and vᵢ = 1/aᵢ + 1/bᵢ + 1/cᵢ + 1/dᵢ. The four cells are events and non-events in RHHR and LHHR. No archived overall-complication row required zero-cell correction. With τ² fixed at 0.026451215, wᵢ = 1/(vᵢ + τ²), Jⱼ = Σwᵢ and Zⱼ = Σwᵢyᵢ/√Jⱼ over studies available at look j.

Two-sided spending follows A(t) = 2Φ[−Φ⁻¹(1−α/2)/√t], capped at t=1. Gaussian quadrature solves successive efficacy boundaries over the preceding continuation region while ignoring futility. Symmetric central futility limits use the corresponding beta-spending function under the target effect; incorrect-direction efficacy stops count toward type-II error. Maximum information is selected numerically so that correct-direction efficacy probability is 0.80 when futility is honored.

Repeated confidence limits at look j are exp(ȳⱼ ± bⱼ/√Jⱼ), where bⱼ is the efficacy boundary. Coverage is conditional on the fixed-parameter independent Gaussian model. These are not stagewise bias-adjusted estimates, equivalence tests, or required independent-patient sample sizes.

| **Archived numerical check** | **OR 0.90** | **OR 0.80** |
| --- | --- | --- |
| Quadrature null error, futility ignored | 0.050000 | 0.050000 |
| Quadrature correct-direction power | 0.800000 | 0.800000 |
| 500,000-sequence null rejection | 5.006% | 5.042% |
| Simulated power, positive / negative | 79.946% / 79.959% | 79.934% / 80.119% |
| Quadrature null error, futility honored | 4.958% | 4.239% |

Archived checks used 240- and 360-node quadrature, a one-look benchmark of 1.959964, an independent two-look bivariate-normal check, and Gaussian-increment simulations (seed 20260924). Archived environment: Python 3.13.5, NumPy 2.3.5, SciPy 1.17.0. The checks refer to the archived execution, not to a new analysis of the clinical data.

Interpretation remains conditional on the archived inputs. The Kooiker overall numerator is a sum of potentially overlapping components; NRD/NIS survey-weighted counts are not independent observed-patient counts; source overlap cannot be excluded. Numerical validation under the model does not establish clinical evidence sufficiency or remove these limitations.

# Supplementary Table S8. Study-selection flow and full-text exclusion reasons

The study-selection counts and exclusion categories correspond to Figure 1 and the eligibility criteria in the main manuscript (19).

| **Stage** | **n** | **Basis** |
| --- | --- | --- |
| Records identified | 592 | 117 PubMed + 338 Embase + 135 Web of Science + 2 Cochrane Library |
| Duplicates removed | 199 | 135 with EndNote + 64 manually |
| Records screened | 393 | 592 − 199 |
| Excluded at title/abstract screening | 344 | As recorded in Figure 1 |
| Reports sought for retrieval | 49 | 393 − 344 |
| Reports not retrieved | 3 | Separate from full-text eligibility exclusions |
| Full-text reports assessed | 46 | 49 − 3 |
| Full-text reports excluded | 35 | Six categories listed below |
| Studies included | 11 | 46 − 35 |

## Reasons for exclusion after full-text assessment

| **Reason** | **Reports** |
| --- | --- |
| Meta-analysis or review | 16 |
| Non-English publication | 1 |
| Conference abstract | 9 |
| Population restricted to patients with obesity | 4 |
| Not a hiatal hernia repair study | 1 |
| Non-comparative study | 4 |
| Total | 35 |

The non-English publication belongs to the 35 full-text exclusions in the confirmed flow, not to the three unretrieved reports. Report-level identities and specific retrieval barriers for the three unretrieved reports are not supplied in the confirmed figure. The aggregate categories above do not constitute a report-by-report exclusion log.

# Supplementary figure legends

Figure S1. Overall postoperative complications: leave-one-out sensitivity estimates for an outcome with original I²=86%. Each row omits the named study; only reduced-data estimates are shown.

Figure S2. Urological complications: leave-one-out sensitivity estimates for original I²=63%. The Soliman omission uses a fixed-effect model after reduced-data I² falls to 33.8%, in accordance with the submitted rule.

Figure S3. Operative time: leave-one-out sensitivity estimates in minutes for original I²=93%. Exclusion of Gerull retains the original fixed-effect MD 22.95 minutes [16.71, 29.19].

Figure S4. Length of stay: leave-one-out sensitivity estimates in days for original I²=97%. Reduced-data point estimates remain negative, but some confidence intervals include zero.

Figure S5. Overall postoperative complications under original-NOS and study-source restrictions. Only the retained 6–8-point NOS scheme is displayed. Removing all large databases gives I²=43.2% and therefore uses the submitted fixed-effect rule.

Figure S6. Power-calibrated information-based sequential assessment for target OR=0.90. Observed cumulative Z values are compared with efficacy and nonbinding futility boundaries. The 100% target is planning-only, with no additional observed study or outcome.

Figure S7. Corresponding sequential assessment for target OR=0.80. The first upper efficacy crossing at Powell is marked. Later attenuation after Ueda is shown retrospectively and does not erase the first crossing. No observed futility entry occurred.

For Figures S1–S5, FE/RE follows the original I²-dependent rule in each reduced dataset; some changes therefore reflect both study omission and model selection. For Figures S6–S7, calibration is conditional on the specified independent Gaussian model and archived nominal variances. These supplementary displays do not replace main Figures 1–11.

# References cited in the supplementary tables

Numbers correspond to the approved main-manuscript reference list; they are not independently renumbered.

19. Page MJ, McKenzie JE, Bossuyt PM, Boutron I, Hoffmann TC, Mulrow CD, et al. The PRISMA 2020 statement: an updated guideline for reporting systematic reviews. BMJ. (2021) 372:n71. doi: 10.1136/bmj.n71.

20. Klock JA, Walters RW, Nandipati KC. Robotic Hiatal Hernia Repair Associated with Higher Morbidity and Readmission Rates Compared to Laparoscopic Repair: 10-Year Analysis from the National Readmissions Database (NRD). J Gastrointest Surg. (2023) 27:489–497. doi: 10.1007/s11605-022-05548-x.

21. Kooiker P, Monnett S, Thompson S, Richmond B. Robotic-assisted versus laparoscopic repair of type II, III and IV hiatal hernias: A retrospective study comparing adverse outcomes. Laparosc Endosc Robot Surg. (2024) 7:11–15. doi: 10.1016/j.lers.2023.12.004.

22. Ward MA, Hasan SS, Sanchez CE, Whitfield EP, Ogola GO, Leeds SG. Complications Following Robotic Hiatal Hernia Repair Are Higher Compared to Laparoscopy. J Gastrointest Surg. (2021) 25:3049–3055. doi: 10.1007/s11605-021-05005-1.

23. Powell C, DeGregorio A, Bews K, Wigle D, Habermann E. Perioperative outcomes of robotic versus laparoscopic paraesophageal hernia repair: a NSQIP analysis. Surg Endosc. (2024) 38:5851–5857. doi: 10.1007/s00464-024-11158-2.

25. Stang A. Critical evaluation of the Newcastle-Ottawa scale for the assessment of the quality of nonrandomized studies in meta-analyses. Eur J Epidemiol. (2010) 25:603–605. doi: 10.1007/s10654-010-9491-z.

26. Wells GA, Shea B, O’Connell D, Peterson J, Welch V, Losos M, et al. The Newcastle–Ottawa Scale (NOS) for assessing the quality of nonrandomised studies in meta-analyses. Ottawa Hospital Research Institute. Available at: https://ohri.ca/en/who-we-are/core-facilities-and-platforms/ottawa-methods-centre/newcastle-ottawa-scale (accessed September 24, 2026).

27. Deeks JJ, Higgins JPT, Altman DG, McKenzie JE, Veroniki AA, editors. Analysing data and undertaking meta-analyses. In: Cochrane Handbook for Systematic Reviews of Interventions, Chapter 10. Updated November 2024. https://www.cochrane.org/authors/handbooks-and-manuals/handbook/current/chapter-10.

28. Wetterslev J, Jakobsen JC, Gluud C. Trial Sequential Analysis in systematic reviews with meta-analysis. BMC Med Res Methodol. (2017) 17:39. doi: 10.1186/s12874-017-0315-7.

29. Lan KKG, DeMets DL. Discrete sequential boundaries for clinical trials. Biometrika. (1983) 70:659–663. doi:10.1093/biomet/70.3.659.

30. Thorlund K, Engstrøm J, Wetterslev J, Brok J, Imberger G, Gluud C. User Manual for Trial Sequential Analysis (TSA). 2nd ed. Copenhagen: Copenhagen Trial Unit (2017). Available at: https://ctu.dk/wp-content/uploads/2021/03/2017-10-10-TSA-Manual-ENG_ER.pdf.

31. Benedix F, Adolf D, Peglow S, Gstettenbauer LM, Croner R. Short-term outcome after robot-assisted hiatal hernia and anti-reflux surgery-is there a benefit for the patient? Langenbecks Arch Surg. (2021) 406:1387–1395. doi: 10.1007/s00423-020-02051-2.

32. Gehrig T, Mehrabi A, Fischer L, Kenngott H, Hinz U, Gutt CN, et al. Robotic-assisted paraesophageal hernia repair-a case-control study. Langenbecks Arch Surg. (2013) 398:691–696. doi: 10.1007/s00423-012-0982-0.

33. Gerull WD, Cho D, Arefanian S, Kushner BS, Awad MM. Favorable peri-operative outcomes observed in paraesophageal hernia repair with robotic approach. Surg Endosc. (2021) 35:3085–3089. doi: 10.1007/s00464-020-07700-7.

34. Lekarczyk A, Sinha H, Dvir D, Goyert J, Airhart A, Reddy RM. Similar hospital profits with robotic-assisted paraesophageal hiatal hernia repair, despite higher or supply costs. Surg Endosc. (2023) 37:3952–3955. doi: 10.1007/s00464-022-09513-2.

35. Soliman BG, Nguyen DT, Chan EY, Chihara RK, Meisenbach LM, Graviss EA, et al. Robot-assisted hiatal hernia repair demonstrates favorable short-term outcomes compared to laparoscopic hiatal hernia repair. Surg Endosc. (2020) 34:2495–2502. doi: 10.1007/s00464-019-07055-8.

36. Wilhelm A, Nocera F, Schneider R, Koechlin L, Daume DL, Fourie L, et al. Robot-assisted vs. laparoscopic repair of complete upside-down stomach hiatal hernia (the RATHER-study): a prospective comparative single center study. Surg Endosc. (2022) 36:480–488. doi: 10.1007/s00464-021-08307-2.

37. Ueda G, Qaraqe T, Han S, Stiles E, Sternback J, Kuppusamy M, et al. Operative trends and clinical outcomes of open, laparoscopic and robotic approaches to hiatal and paraoesophageal hernias- a study of 1834 patients. J Robot Surg. (2025) 19:145. doi: 10.1007/s11701-025-02299-0.
