# Supplementary material for: Perioperative safety profile of robotic versus laparoscopic hiatal hernia repair: a systematic review and meta-analysis
Source: Front Surg. 2026 Oct 21;13:1993235. doi: 10.3389/fsurg.2026.1993235 (PMC13731218; doi:10.3389/fsurg.2026.1993235)
Supplement: Supplementary file 2 [file Table2.docx]

# Supplementary Material 2. Database-specific search strategies

Perioperative safety profile of robotic versus laparoscopic hiatal hernia repair: a systematic review and meta-analysis

The publication-eligibility interval in the main manuscript is 1 January 2010 through 15 July 2025. The original search is reported there as completed on 15 July 2025. The PubMed expression below is the manuscript expression; Embase combines the recovered original blocks. The Web of Science and Cochrane expressions were verified by the authors during revision and are distinguished from the original searches.

| **Database** | **Historical export** | **Records** |
| --- | --- | --- |
| MEDLINE via PubMed | pubmed-17and9-set.nbib | 117 |
| Embase | records.ris | 338 |
| Web of Science | savedrecs.ciw | 135 |
| Cochrane Library | citation-export.ris | 2 |

These are historical exported-record counts, not reported yields of a new validation run. Exact revision-verification dates, platform collections/editions and interface limits are not documented in the materials supplied for this supplement. Publication eligibility is not assumed to prove which interface filters were enabled.

## PubMed / MEDLINE

(("hernia, hiatal"[MeSH Terms]) OR ("Herni*, Hiat*"[Title/Abstract]) OR ("Hiat* Herni*"[Title/Abstract]) OR ("Paraesophageal Hiatal Hernia*"[Title/Abstract]) OR ("Sliding Hiatal Hernia*"[Title/Abstract]) OR ("Paraesophageal Hernia*"[Title/Abstract]) OR ("Sliding Esophageal Hernia"[Title/Abstract]) OR ("Esophageal Hernia*"[Title/Abstract]) OR ("Hernia*, Esophageal"[Title/Abstract])) AND (("Robotic Surgical Procedures"[MeSH Terms]) OR ("Robotic Surgical Procedure*"[Title/Abstract]) OR ("Robotic-Assisted Surger*"[Title/Abstract]) OR ("Robot-Assisted Surger*"[Title/Abstract]) OR ("Robot Surger*"[Title/Abstract]) OR ("Robot-Enhanced Procedure*"[Title/Abstract]))

# Embase.com

#1 'hiatal hernias':ti,ab,kw OR 'hiatus hernia':ti,ab,kw OR 'sliding esophageal hernia':ti,ab,kw OR 'esophageal hernia':ti,ab,kw OR 'hernia, esophageal':ti,ab,kw OR 'hiatus hernia'/exp

#2 'surgical procedures, robotic':ti,ab,kw OR 'robot-enhanced procedures':ti,ab,kw OR 'robot surgery':ti,ab,kw OR 'robotic-assisted surgeries':ti,ab,kw OR 'procedure, robotic surgical':ti,ab,kw OR 'robot assisted surgery'/exp

#3 #1 AND #2

## Expanded equivalent expression

('hiatal hernias':ti,ab,kw OR 'hiatus hernia':ti,ab,kw OR 'sliding esophageal hernia':ti,ab,kw OR 'esophageal hernia':ti,ab,kw OR 'hernia, esophageal':ti,ab,kw OR 'hiatus hernia'/exp) AND ('surgical procedures, robotic':ti,ab,kw OR 'robot-enhanced procedures':ti,ab,kw OR 'robot surgery':ti,ab,kw OR 'robotic-assisted surgeries':ti,ab,kw OR 'procedure, robotic surgical':ti,ab,kw OR 'robot assisted surgery'/exp)

# Web of Science

TS=("hiatal hernia" OR "herni* hiat*" OR "hiat* herni*" OR "paraesophageal hiatal hernia*" OR "sliding hiatal hernia*" OR "paraesophageal hernia*" OR "sliding esophageal hernia" OR "esophageal hernia*" OR "hernia* esophageal") AND TS=(robot*)

The TS=(robot*) expression is the author-verified revision version reproduced in the manuscript; it is not presented as a recovered historical query log.

# Cochrane Library: Search Manager

#1 MeSH descriptor: [Hernia, Hiatal] explode all trees

#2 ((herni* NEXT hiat*) OR (hiat* NEXT herni*) OR (paraesophageal NEXT hiatal NEXT hernia*) OR (sliding NEXT hiatal NEXT hernia*) OR (paraesophageal NEXT hernia*) OR (sliding NEXT esophageal NEXT hernia) OR (esophageal NEXT hernia*) OR (hernia* NEXT esophageal)):ti,ab,kw

#3 MeSH descriptor: [Robotic Surgical Procedures] explode all trees

#4 ((robotic NEXT surgical NEXT procedure*) OR (robotic NEXT assisted NEXT surger*) OR (robot NEXT assisted NEXT surger*) OR (robot NEXT surger*) OR (robot NEXT enhanced NEXT procedure*)):ti,ab,kw

#5 (#1 OR #2) AND (#3 OR #4)

Each #n denotes a search-history line. The two archived Cochrane citations link to CENTRAL; that establishes the origin of those results, but does not by itself establish which collections were selected when the original query was run.
