# Supplementary material for: Perioperative safety profile of robotic versus laparoscopic hiatal hernia repair: a systematic review and meta-analysis
Source: Front Surg. 2026 Oct 21;13:1993235. doi: 10.3389/fsurg.2026.1993235 (PMC13731218; doi:10.3389/fsurg.2026.1993235)
Supplement: Supplementary file 3 [file Table3.docx]

# PRISMA 2020 checklist

02_Manuscript_FRONTIERS_FORMAT_POLISHED_CLEAN.docx. Entries report what is present; incomplete reporting is not relabeled as not applicable.

| **Item** | **Section/topic** | **Checklist item** | **Location / status** |
| --- | --- | --- | --- |
| 1 | Title | Identify the report as a systematic review. | Title, p. 1. |
| 2 | Abstract | See the PRISMA 2020 for Abstracts checklist. | Structured abstract, p. 1. |
| 3 | Rationale | Describe the rationale for the review in the context of existing knowledge. | Introduction, p. 2. |
| 4 | Objectives | Provide an explicit statement of the objective(s) or question(s) the review addresses. | Introduction, p. 2; Eligibility criteria, pp. 2–3. |
| 5 | Eligibility criteria | Specify the inclusion and exclusion criteria for the review and how studies were grouped for the syntheses. | Eligibility criteria, pp. 2–3; Table S8. |
| 6 | Information sources | Specify all databases, registers, websites, organisations, reference lists and other sources searched or consulted to identify studies. Specify the date when each source was last searched or consulted. | Search strategy, pp. 3–4; Supplementary Material 2. Exact revision-verification dates/collections are not documented. |
| 7 | Search strategy | Present the full search strategies for all databases, registers and websites, including any filters and limits used. | Search expressions, pp. 3–4; Supplementary Material 2. Platform-specific limits are not documented. |

# PRISMA 2020 checklist (continued)

| **Item** | **Section/topic** | **Checklist item** | **Location / status** |
| --- | --- | --- | --- |
| 8 | Selection process | Specify the methods used to decide whether a study met the inclusion criteria of the review, including how many reviewers screened each record and each report retrieved, whether they worked independently, and if applicable, details of automation tools used in the process. | Study selection, p. 4. |
| 9 | Data collection process | Specify the methods used to collect data from reports, including how many reviewers collected data from each report, whether they worked independently, any processes for obtaining or confirming data from study investigators, and if applicable, details of automation tools used in the process. | Data extraction, p. 4. |
| 10a | Data items | List and define all outcomes for which data were sought. Specify whether all results that were compatible with each outcome domain in each study were sought (e.g. for all measures, time points, analyses), and if not, the methods used to decide which results to collect. | Outcome definitions and analysis denominators, pp. 4–5; Table S2. |
| 10b | Data items | List and define all other variables for which data were sought (e.g. participant and intervention characteristics, funding sources). Describe any assumptions made about any missing or unclear information. | Data extraction, p. 4; main Tables 1–3, pp. 16–18. |
| 11 | Study risk of bias assessment | Specify the methods used to assess risk of bias in the included studies, including details of the tool(s) used, how many reviewers assessed each study and whether they worked independently, and if applicable, details of automation tools used in the process. | Risk of bias assessment, p. 5; main Table 4, p. 19; Table S3. Expanded guidance does not certify new independent verification. |
| 12 | Effect measures | Specify for each outcome the effect measure(s) (e.g. risk ratio, mean difference) used in the synthesis or presentation of results. | Statistical analysis, p. 5; Tables S4–S7. |

# PRISMA 2020 checklist (continued)

| **Item** | **Section/topic** | **Checklist item** | **Location / status** |
| --- | --- | --- | --- |
| 13a | Synthesis methods | Describe the processes used to decide which studies were eligible for each synthesis (e.g. tabulating the study intervention characteristics and comparing against the planned groups for each synthesis (item #5)). | Eligibility and outcome definitions, pp. 2–5; Tables S2, S4–S5. |
| 13b | Synthesis methods | Describe any methods required to prepare the data for presentation or synthesis, such as handling of missing summary statistics, or data conversions. | Statistical analysis, pp. 5–6; S4 and the supplied analysis code. Preparation/conversion of all original continuous data is not fully detailed. |
| 13c | Synthesis methods | Describe any methods used to tabulate or visually display results of individual studies and syntheses. | Statistical analysis, p. 5; main Figures 2–11 and supplementary Figures S1–S7. |
| 13d | Synthesis methods | Describe any methods used to synthesize results and provide a rationale for the choice(s). If meta-analysis was performed, describe the model(s), method(s) to identify the presence and extent of statistical heterogeneity, and software package(s) used. | Statistical and sequential analysis, pp. 5–6; Tables S4–S7; code archive. |
| 13e | Synthesis methods | Describe any methods used to explore possible causes of heterogeneity among study results (e.g. subgroup analysis, meta-regression). | Sensitivity analyses and meta-regression, pp. 5–6; results, p. 9; Tables S4–S6. |
| 13f | Synthesis methods | Describe any sensitivity analyses conducted to assess robustness of the synthesized results. | Sensitivity analyses, pp. 5–6; results, p. 9; Tables S4–S5 and Figures S1–S5. |
| 14 | Reporting bias assessment | Describe any methods used to assess risk of bias due to missing results in a synthesis (arising from reporting biases). | No dedicated formal reporting-bias assessment method is reported. GRADE considers publication bias (p. 5); this does not replace a reporting-bias analysis. |
| 15 | Certainty assessment | Describe any methods used to assess certainty (or confidence) in the body of evidence for an outcome. | Certainty of evidence, p. 5; Table S1. |

# PRISMA 2020 checklist (continued)

| **Item** | **Section/topic** | **Checklist item** | **Location / status** |
| --- | --- | --- | --- |
| 16a | Study selection | Describe the results of the search and selection process, from the number of records identified in the search to the number of studies included in the review, ideally using a flow diagram. | Study selection, p. 7; Figure 1; Table S8. |
| 16b | Study selection | Cite studies that might appear to meet the inclusion criteria, but which were excluded, and explain why they were excluded. | Aggregate reasons: p. 7 and Table S8. Report-by-report citations and specific retrieval barriers are not supplied. |
| 17 | Study characteristics | Cite each included study and present its characteristics. | Study characteristics, p. 7; main Tables 1–3, pp. 16–18. |
| 18 | Risk of bias in studies | Present assessments of risk of bias for each included study. | Risk of bias, p. 7; main Table 4, p. 19; Table S3. |
| 19 | Results of individual studies | For all outcomes, present, for each study: (a) summary statistics for each group (where appropriate) and (b) an effect estimate and its precision (e.g. confidence/credible interval), ideally using structured tables or plots. | Main Figures 2–11; supplied archived analysis inputs for the supplementary computations. |
| 20a | Results of syntheses | For each synthesis, briefly summarise the characteristics and risk of bias among contributing studies. | Results, pp. 7–9; Tables S1–S7. |
| 20b | Results of syntheses | Present results of all statistical syntheses conducted. If meta-analysis was done, present for each the summary estimate and its precision (e.g. confidence/credible interval) and measures of statistical heterogeneity. If comparing groups, describe the direction of the effect. | Primary and secondary results, pp. 7–9; main Figures 2–11. |

# PRISMA 2020 checklist (continued)

| **Item** | **Section/topic** | **Checklist item** | **Location / status** |
| --- | --- | --- | --- |
| 20c | Results of syntheses | Present results of all investigations of possible causes of heterogeneity among study results. | Supplementary results, p. 9; Tables S4–S6; Figures S1–S5. |
| 20d | Results of syntheses | Present results of all sensitivity analyses conducted to assess the robustness of the synthesized results. | Supplementary results, p. 9; Tables S4–S5; Figures S1–S5. |
| 21 | Reporting biases | Present assessments of risk of bias due to missing results (arising from reporting biases) for each synthesis assessed. | No dedicated formal reporting-bias result is reported. Table S1 records the retained GRADE discussion of publication bias. |
| 22 | Certainty of evidence | Present assessments of certainty (or confidence) in the body of evidence for each outcome assessed. | Risk of bias / certainty results, p. 7; Table S1. |
| 23a | Discussion | Provide a general interpretation of the results in the context of other evidence. | Discussion, pp. 9–12. |
| 23b | Discussion | Discuss any limitations of the evidence included in the review. | Limitations, pp. 11–12; Tables S2, S3 and S7. |
| 23c | Discussion | Discuss any limitations of the review processes used. | Limitations, pp. 11–12; search-documentation gaps in Supplementary Material 2. |
| 23d | Discussion | Discuss implications of the results for practice, policy, and future research. | Clinical implications and Conclusions, pp. 11–12. |

# PRISMA 2020 checklist (continued)

| **Item** | **Section/topic** | **Checklist item** | **Location / status** |
| --- | --- | --- | --- |
| 24a | Registration and protocol | Provide registration information for the review, including register name and registration number, or state that the review was not registered. | Protocol and reporting standard, p. 2: PROSPERO CRD420251102047. |
| 24b | Registration and protocol | Indicate where the review protocol can be accessed, or state that a protocol was not prepared. | Registration number is reported, p. 2. The protocol itself is not included in this attachment set. |
| 24c | Registration and protocol | Describe and explain any amendments to information provided at registration or in the protocol. | Additional analyses are described as post hoc, pp. 5–6. A complete protocol-amendment log is not supplied. |
| 25 | Support | Describe sources of financial or non-financial support for the review, and the role of the funders or sponsors in the review. | Funding, p. 12. |
| 26 | Competing interests | Declare any competing interests of review authors. | Conflict of interest, p. 12. |
| 27 | Availability of data, code and other materials | Report which of the following are publicly available and where they can be found: template data collection forms; data extracted from included studies; data used for all analyses; analytic code; any other materials used in the review. | Data availability, p. 12; accompanying code, archived inputs and results. Complete original screening and extraction materials are not all included. |

19. Page MJ, McKenzie JE, Bossuyt PM, Boutron I, Hoffmann TC, Mulrow CD, et al. The PRISMA 2020 statement: an updated guideline for reporting systematic reviews. BMJ. (2021) 372:n71. doi: 10.1136/bmj.n71. Checklist wording retained from the supplied PRISMA 2020 template.
